# Physical and Mechanical Properties of Natural Leaf Fiber-Reinforced Epoxy Polyester Composites

**DOI:** 10.3390/polym13091369

**Published:** 2021-04-22

**Authors:** Sanjeev Kumar, Lalta Prasad, Vinay Kumar Patel, Virendra Kumar, Anil Kumar, Anshul Yadav, Jerzy Winczek

**Affiliations:** 1 Department of Mechanical Engineering, GB Pant Institute of Engineering and Technology, Pauri Garhwal 246194, India; sanjeevkumar001.1991@gmail.com (S.K.); vinaykrpatel@gmail.com (V.K.P.); 2 Department of Mechanical Engineering, National Institute of Technology Uttarakhand, Srinagar 246174, India; laltaprasad@nituk.ac.in; 3 Department of Mechanical Engineering, Kamla Nehru Institute of Technology, Sultanpur 228118, India; veer.iitdmech@gmail.com (V.K.); anilk@knit.ac.in (A.K.); 4 Membrane Science and Separation Technology Division, CSIR-Central Salt and Marine Chemicals Research Institute, Bhavnagar 364002, India; anshuly@csmcri.res.in; 5 Faculty of Mechanical Engineering and Computer Science, Częstochowa University of Technology, 42-201 Częstochowa, Poland

**Keywords:** thermoset polymer, natural composite, environment sustainability, chemical treatment, mechanical properties

## Abstract

In recent times, demand for light weight and high strength materials fabricated from natural fibres has increased tremendously. The use of natural fibres has rapidly increased due to their high availability, low density, and renewable capability over synthetic fibre. Natural leaf fibres are easy to extract from the plant (retting process is easy), which offers high stiffness, less energy consumption, less health risk, environment friendly, and better insulation property than the synthetic fibre-based composite. Natural leaf fibre composites have low machining wear with low cost and excellent performance in engineering applications, and hence established as superior reinforcing materials compared to other plant fibres. In this review, the physical and mechanical properties of different natural leaf fibre-based composites are addressed. The influences of fibre loading and fibre length on mechanical properties are discussed for different matrices-based composite materials. The surface modifications of natural fibre also play a crucial role in improving physical and mechanical properties regarding composite materials due to improved fibre/matrix adhesion. Additionally, the present review also deals with the effect of silane-treated leaf fibre-reinforced thermoset composite, which play an important role in enhancing the mechanical and physical properties of the composites.

## 1. Introduction

The evolution of various efficient natural fibres as reinforcing material made natural fibre polymer composites (NFPC’s) a key attraction for the couple of decades. In the present scenario, scientists and technologists have highlighted natural fibres supremacy over synthetic fibres as reinforcement materials. Synthetic fibres (for e.g. glass fibre and carbon fibre, etc.) were used earlier in polymer composite because of their high mechanical strength in various engineering applications. In view of environmental concern, the synthetic fibre polymer composite materials are not eco-friendly and non-biodegradable. However, when natural fibre is introduced in the field as a replacement for synthetic fibre, many studies have reported that natural fibre-reinforced polymer composite sustainability is high [1,2,3,4,5,6,7,8,9]. Its acceptability to the environment is better due to renewability and biodegradability. Nowadays, researchers are developing the governing acts and general social perception regarding energy, raw material wastage, and pollution towards the environment. The issue can be minimized by using natural fibres. Ancient civilizations were involved in using natural fibres in various forms to meet their requirements. As time passed, the fibre extraction from the plant became significant and easy due to advancement in technology. 

**Figure 1:**
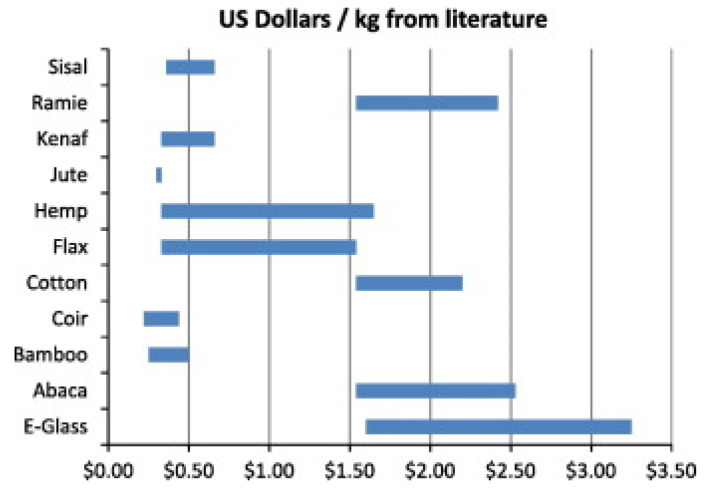
Cost per kg weight comparison between glass and natural fibres. Reprinted with the permission from Reference [14]. Copyright 2012 Elsevier.

NFPC’s and their utilization have increased tremendously in various engineering applications like automobile, packaging, structural component, aerospace, marine, electronic industry, sports goods, microbial fuel cell, air filter, and housing panels. The NFPC’s showed high strength due to the presence of the cellulosic component. Recently, industries found that natural fibres have great potential in the outdoor insulation, production of nano-whisker and packaging applications [9]. NFPC’s demonstrated several advantages such as high specific strength, wide availability, low density, renewability, high stiffness, a high degree of flexibility, less energy expenditure, low health risk, and low abrasiveness. NFPC’s are biodegradable, inexpensive, and own excellent mechanical performance, which made them a favorite attraction for many industries [10]. NFPC’s also offers disadvantages to some extentas natural fibres are hydrophilic, increased water intake while matrices are hydrophobic (water-resistant) which decreases the adhesion behaviour between fibre and matrices causing a reduction in their physical and mechanical properties [11]. However, various treatment methods of natural fibres increase the fibre/matrix adhesion as reported in literature for enhancing the adhesion in fibre/polymer composite material [12,13]. The cost comparison is an essential factor, since natural fibres have much lower density and strength than glass fibres. Cost per weight comparison between glass and natural fibres is shown in Figure 1 [14]. The figure clearly states that natural fibres, mainly bamboo, sisal, jute, kenaf, are useful to compete with glass fibres. Overall, it was found that lower densities of natural fibres are cheap compared to glass fibres [14].

Bio-composites materials have played a promising role in their substituting petrochemical materials with materials having less greenhouse gas emissions [15]. Natural fibre composites usage would provide an alternative to minimize carbon footprint due to the capability of carbon dioxide (CO_2_) absorption by natural fibre resources [16,17,18]. 

## 2. Environmental Factor for Sustainability

**Figure 2:**
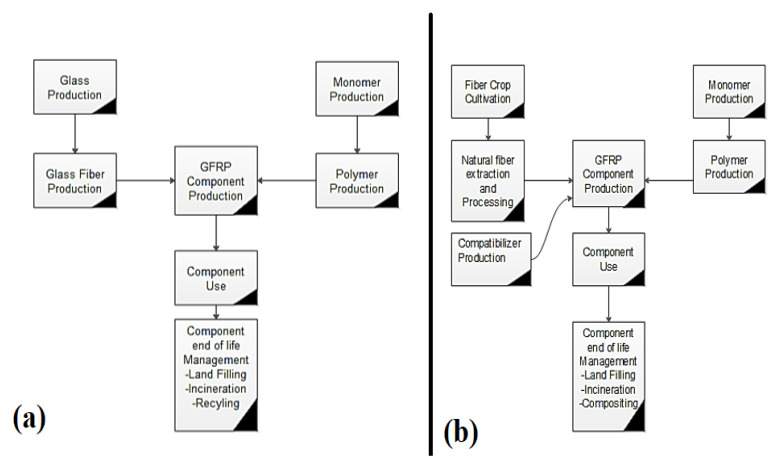
(**a**) Life cycle stages of glass fibre-reinforced polymer (GFRP) composite, and (**b**) Life cycle stages of natural fibre-reinforced polymer composite component. Adapted with the permission from Reference [20]. Copyright 2004 Elsevier.

**Figure 3:**
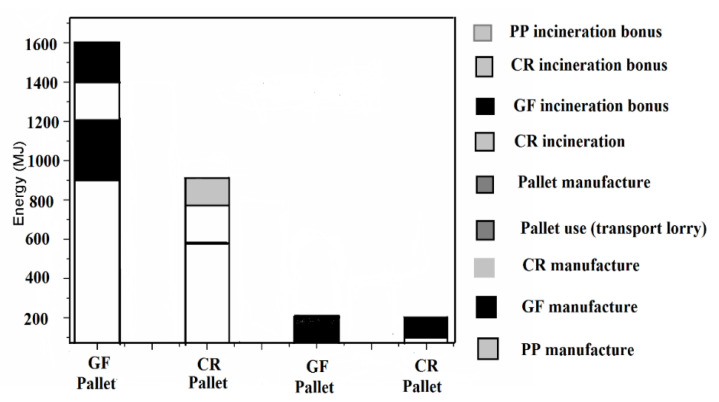
Life cycle assessment (LCA) study for energy consumption for the glass fibre (GF) and China reed (CR) pallets. Reprinted with the permission from Reference [21]. Copyright 2001 Elsevier.

**Figure 4:**
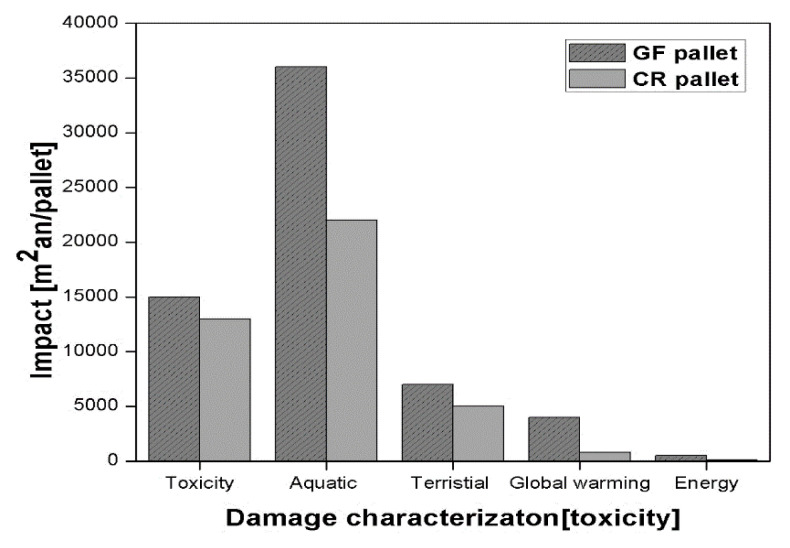
Critical surface time study for damage characterization of glass fibre (GF) and China reed (CR) pallet. Reprinted with the permission from Reference [21]. Copyright 2001 Elsevier.

The glass fibre (GF) polymer composite used in automobile applications have been reported by Zini et al. [19]. They found that disposal of composite materials is a severe problem at the end of the life cycle process. During incineration, about 50% of the total volume remains as a residue (unburned). The use of glass fibre reinforced polymer (GFRP) composite material causes environmental pollution if thrown in the open atmosphere. Another disadvantage is that the specific weight of GFRP composite material is high as compared to natural fibre polymer composite materials. The cultivation of the fibrous plant is dependent on sunlight (solar energy). The production, extraction, and processing of natural fibre from the plant is easier than synthetic fibre. The conventional fuel (fossil fuel) required for natural fibre processing is about 10% lower than that of synthetic fibre [19]. The emission of pollutants (especially gases) is on a higher side for synthetic fibre than natural fibre as reported by Begum et al. [12]. A study on life cycle assessment (LCA) for GFRP and natural fibres reinforced polymer was reported by Joshi et al. [20], as shown in Figure 2 [20]. Another study of LCA on GF and china reed (CR) fibre pallets reinforcing polypropylene (PP) has been reported by Nicollier et al. [21]. The LCA consists of GF and CR fibre extraction, pallets production, transportation, disposal, its use, and incineration of the composite. It is clear from Figure 3 [21], that the CR fibre pallets have many advantages over the GF pallets, such as less fuel consumption during transportation (due to less weight), non-toxic, and low effect on global warming. The emissions of pollutants (say heavy metals) from the CR fiber are lesser than the GF and higher lifetime according to the critical surface time method (CST95) as shown in Figure 4 [21].

**Figure 5:**
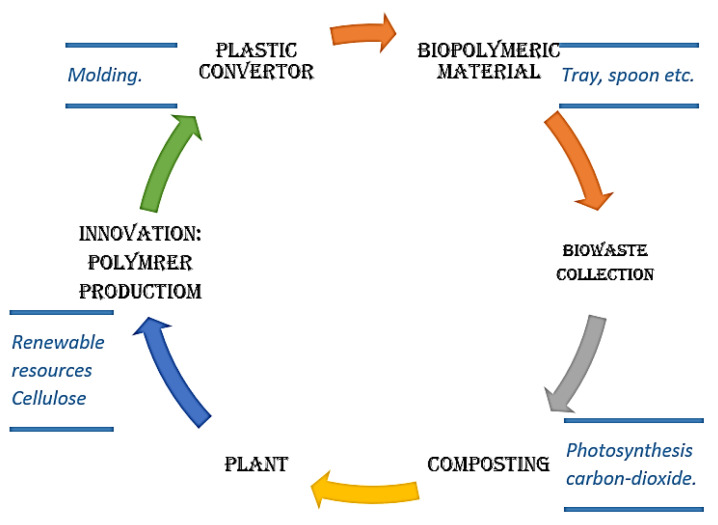
Carbon dioxide (CO_2_) sequestration for composite fabricated from renewable source.

Haylock et al. [22] reported the behavior of composites using bio-based and conventional fillers in polylactic acid (PLA). The study observed that bio-based fillers in PLA composites are a feasible and cost-effective method of production. Also, the use of organic filler decreases various environmental difficulties compared to manufacturing inorganic filler-based composite. The utilization of organic bio-based fillers gives advantages to recycling, the end of life cycle process, and landfilling. Bio-based fillers are comparable or superior performance as compared to common petroleum-based plastics [22]. LCA of new vehicle technologies have been performed, and the outcomes depend significantly on the methodologies and assumptions used in performing an LCA on products, such as automobiles can be very expensive, more time-consuming, and complicated. LCA is a useful tool for compiling the inputs and outputs associated with the life cycle products/services to evaluate any potential impacts [23]. The sustainability of bio-composite is high, and its acceptability to the environment is better due to renewability and biodegradability. The composite materials fabricated from renewable sources maintain carbon dioxide neutrality, as shown in Figure 5 [24].

## 3. Classification of Natural Leaf Fibers

**Figure 6:**
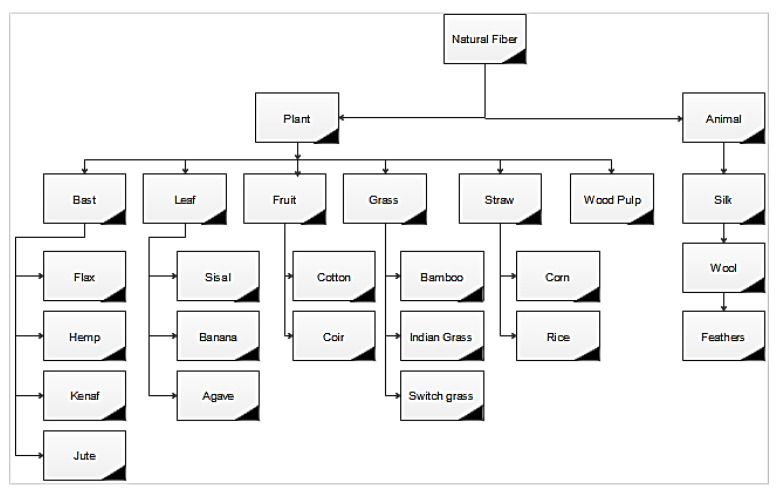
Classification of natural fibre. Adapted with the permission from Reference [19]. Copyright 2011 John Wiley and Sons.

The natural fibre is classified into two forms namely, plant fibre and animal fibre. Natural fibre can be extracted from plants and classified according to their utilization. The fibre is directly extracted from the primary plant, and fibre is produced as a by-product called secondary plant [25]. A broad classification of the natural fibres is shown in Figure 6, as reported by Zini et al. [19]. The primary fibres are jute, hemp, Kenaf, sisal, and secondary fibres are pineapple, oil palm, and coir. The leaf fibers are a group of long, multiple-celled lignocellulosic fibers extracted from plants, generally, organize as leafage. While leaves can be diversified in appearance and function, the leaf that can be used for the production of fiber tends to be short and long, with limited structures that can be mechanically detached with relative comfort. The leaf of different lignocellulosic plants that are generally used as reinforcement for polymer composites material comprises those of sisal, agave, banana, pineapple, abaca, and date palm fiber [26,27,28]. Sisal leaf fiber, also known as agave sisalana, is commonly produced in tropical countries, e.g., Brazil and East Africa. Its life span is about 7–10 years, with 200–250 leaves, and each of them extracts about 1000 bundles of fiber [29]. The sisal plant generally grows up to 2 m; sisal fiber length varies according to climatic condition, i.e., 6–1.5 m. The leaf of the sisal plant is enclosed with three categories of fiber: structural, arch, and xylem [10]. Among three, the structural fiber is the most important type, due to non-split nature at the time of extraction, arch fiber has the ability to hold high mechanical strength and hence use in short fiber application. However, xylem fiber is lost at the time of extraction. Sisal fiber has a dominant fiber due to its strength, endurance, stretch ability, and resistance ability to degradation in saltwater. However, as the temperature increases the mechanical strength, toughness, and modulus is decreases [30]. The overall requirement for sisal lignocellulosic fibre and its commodity somehow decreased in years 1998–2000 and 2010 by a yearly rate of 2.3% in terms of agricultural yarn. Agave fiber belongs to the same family of sisal plant fiber. It is extracted from basal part of agave plant, length of fiber is about 0.5–2.2 m long, thick, flat, and margins outwardly directed spines. It is generally used to make ropes, mats, and twines in rural areas. It generally grows in dry uncovered waste lands mostly above 1300 m. It is generally found in the north and south region of India and its native plant is also found in North America.

Banana is the largest growing aromatic flowering plant. The leaves of the banana plant are arranged spirally and can grow up to 2.0–2.7 m long. They are usually used as eco-friendly biodegradable plates and food containers in most Asian countries. The chemical constituent such as cellulose in banana and sisal lignocellulosic fiber is almost the same, but the structure of the banana fiber is different than sisal fiber, i.e., the spiral angle is 11° in a banana and 20° sisal [31]. Therefore, the mechanical strength of the banana fiber is higher than sisal [32]. Pineapple fiber is extracted from the leaves of the pineapple plant, and it is found generally in Asia and the Philippines. It is mixed with polyester or silk to produced fabric for textiles, mops, curtains, and carpets. The pineapple plant spreads all over the tropical countries as fruit, and thus pineapple fiber is treated as an agricultural by-product. The leaves of the pineapple plant are long, about 1 m, but the crop of the pineapple plant has a short stem. The chemical content present in pineapple fiber is identical to flax fiber, and the structural angle is comparably lower (14°). The mechanical strength of pineapple fiber is similar as compared to jute fibers, but the fiber structure of pineapple has no mesh. Pineapple fibre is highly microscopic, and the fiber strength can reduce by 50% when pineapple fibers are wet [33]. Abaca plant belongs to indigenous banana species generally produced in the countries like the Philippines. The leaf height of the abaca plant is about 3–4 m. Fiber is extracted from the leaf wrap of the abaca plant, also known as “manila hemp” even though it does not belong to bast fiber. It is usually used for ropes and twines. It is chosen as the strongest among all lignocellulosic plant fibers because its mechanical strength is three times more than cotton and two times better more than the sisal fiber. Additionally, it has a superior capability to resist salt water [10]. Date Palm fiber is cultivated from sweet palm fruit-growing trees native to Northern Africa, India, Middle East, India, United States (US), and the Canary Islands. The general palm tree leaf is 3–5 m long. The stem part of the palm tree is wrapped in a single fiber with mesh. Usually, these fibers are mostly used to make baskets and ropes [10].

## 4. Chemical Constituent of Natural Fibre

The main constituents in natural fibres are mainly three polymers such as cellulose (60–80%), hemicelluloses, amorphous lignin, and moisture up to 5–20% [26]. Apart from these constituents, the natural fibre also contains pectin, wax, protein, tannins, inorganic salt, and water-soluble compound, depending upon the climatic condition. The climatic conditions have a significant effect on the polymers. The amount of these polymers and extractives vary from plant to plant [24]. The plant contains cellulose, which is a primary structure of the cell wall. Cellulose has a crystalline structure that gives high strength to the plant. Cellulose has a long polymer chain with a high degree of polymerization (≤10,000) and high molecular weight (<500,000) [24,34,35]. Plants have another cell wall known as hemicellulose. Hemicellulose has a random unshaped structure that gives low strength, low degree of polymerization (<100–200), and highly hydrophilic (water intake) in nature. Simultaneously, lignin is a complex branched polymer of phenyl propane and is an important part of plant’s secondary cell walls. Lignin acts as a bonding agent for cellulose fibres that hold neighbour cells together, and its thermal durability is better than cellulose and hemicellulose [35,36].

**Figure 7:**
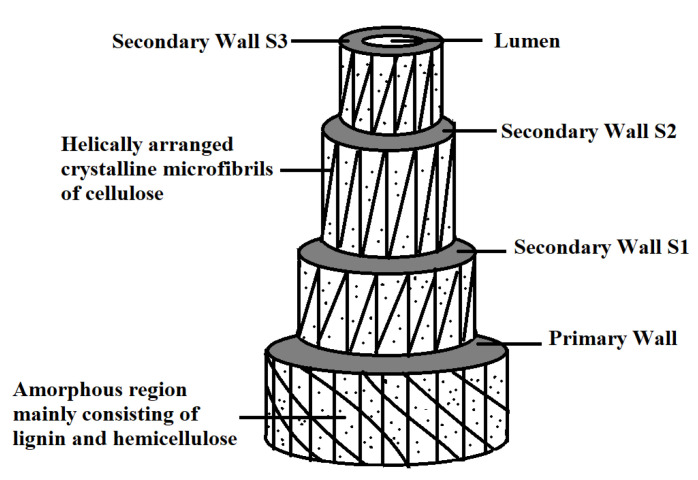
The structure of a single fibre cell.

Fiber extracted from plants such as bast, leaves, and grasses is a bundle of single fibers consisting of many fiber divisions. A single fiber can be treated as a hollow composite. Cellulose present in the fiber behaves like a reinforcing material. On the other hand, non-cellulosic components (hemicellulose, lignin, waxes, inorganic salt, and other content) helps the matrix to hold the fiber together. Here are different layers present in a single fiber as shown in Figure 7 [10], the lumen present in the center, secondary wall (S3, S2, and S1), and primary wall located from inside to outwards. Cellulose and hemicellulose accumulate in the primary layer, i.e., the first layer of fiber, whose growth encircles the secondary wall [37]. The secondary wall contains helically arranged crystalline microfiber of cellulose in the amount of 30–100 molecules and has a diameter of 10–30nm, which provides mechanical strength to the fiber. The S1 and S3 layers are thin as compared to the S1 layer, and the S1 layer shares near about 70% of fibre Young’s modulus. The spiral angle between fiber axis and micro-fibre is mainly dependent on the plant species. Layer S2 and spiral angle play a dominant role in the mechanical properties of the fiber, i.e., the smaller the spiral angle higher the fiber strength and modulus [38]. Middle lamella, also known as the outer layer, contains lignin and pectin to hold the fiber bundle together to construct the final lignocellulosic fiber structure. The presence of lignin and pectin decreases the mechanical strength and interface properties between matrix and fiber. The chemical composition of different natural fibre is presented in Table 1.

## 5. Matrices of Bio-Composite

Natural fibres play a crucial role in polymer science as sustainable reinforcing materials in a different polymeric resin. Matrices for bio-composite are generally thermoset and thermoplastic; they play a crucial role in deciding the resulting composite’s physical and mechanical behaviour. Thermoset composite is used with natural fibre for developing a wide variety of strength performance. Thermoset polymers are polyester, epoxy, and vinyl ester resin. Thermoset composites are challenging to set up due to catalysts, hardeners, flowing agents, and curing agents. Chemically, the thermoset polymer is maintained as a 3D network structure, highly cross-linked, rigid, creep resistant, and highly opposed to solvent [2]. The surface modification has much more significance in the improvement of the performance of thermoset composite. Cellulosic fibres without surface modification show weaker properties than human-made fibres like E-glass and carbon fibre. Thermoset composites are not very strong as traditional-reinforced systems (human-made fibre composite). The lower density of natural fibres compared to human-made like E-glass makes it a practical consideration for high specific strength reinforcing material, as reported by Fuqua et al. [10].

Thermoplastic polymers are polyethylene (PE), high-density polyethylene (HDPE), low-density polyethylene (LDPE), polypropylene (PP), polyvinyl chloride (PVC), and polystyrene (PS). They are formulated by simple methods (extrusion and injection molding) as compared to thermoset polymer [35]. In thermoplastic composite, polyethylene (PE) is favored because it has less density, good electrical insulation, dimensionally stable, and good impact property. Thermoplastics are flexible, tough, require low surface modification, a limited percentage of loading, and show good mechanical properties.

The major problems associated with thermoplastic natural fiber composite are strongly affected by temperature, processing time, matrix interface, and dispersion between fibre and polymers. The thermal degradation of natural fibre (lignocellulosic fiber) takes place around 180–210 °C. Therefore, the processing of composite made up of thermoplastic must be temperature limited. Processing time is limited for the degradation of natural fiber at low temperatures. Otherwise, it damages the fiber significantly and reduces the composite’s performance. However, thermoplastic natural fiber composite strength is improved by using compatibilizers like maleated ethylene, maleated propylene, and acrylic-grafted linear polymer. The enhancement of mechanical properties is due to coupling reaction between cellulose and hydroxyl group, enhancing the adhesion between fiber/matrix [23]. Table 2 and Table 3 show the general characteristics of thermoset polymer and thermoplastic polymer.

## 6. Method of Fabrication of Composites

The demand for robust, durable, and lightweight materials is growing tremendously. Most industries are turning to composites with different manufacturing techniques. Depending on materials, design, and application, various composite fabrication methods are available in the industry. Manufacturing of natural composites involves manufacturing fibre preform and reinforcing these fibres in the matrix material using various methods. Fibre preforms involve knitting, weaving, trimming, and stitching fibres in long sheets or mat structures [45,46,47].

### 6.1. Conventional Manufacturing Processes

The most basic manufacturing method for thermoset composites is hand layup, and a roller is used to press the resin into the fabrics to ensure proper interaction between the successive layers of the filler and matrix [48,49,50]. The small modification in the hand layup method is converted into a spray-up technique. A handgun is used to sprays resin and sliced fibres on a mould. Simultaneously, a roller is used to merge these fibres into the matrix material [51,52]. Vacuum bag moulding is an extension of the wet layup process and uses a flexible film made of nylon polyethene or polyvinyl alcohol (PVA). Many times, the vacuum bag moulding technique is performed with the help of the hand layup technique. Laminate is first made using the hand layup technique, and then after, it is placed between the vacuum bag and the mould to ensure fair infusion of fibres into the matrix material [53,54]. Resin infusion processes are fast and automated hand layup methods to meet the increasing demand for composite materials. Vacuum infusion or vacuum-assisted resin transfer moulding (VARTM) is a recent development in which preform fibres are placed on a mould, and a perforated tube is positioned between the vacuum bag and resin trunk. The vacuum force causes the resin to be sucked through the perforated tubes over the fibres to consolidate the laminate structure. This process leaves no space for excess air in the composite structure, making it famous for producing large objects like boat hulls and wind turbine blades [55,56]. Compression moulding is a high-volume thermoset moulding process that employs costly but extremely durable metal dies. It offers a concise cycle time, a great degree of productivity, and automation throughout dimensional stability; therefore, it finds various automobile industry applications [57,58,59]. The pultrusion process is a continuous process, useful for the fabrication of composites with a constant cross-section with a relatively long length. In this process, fibres are pulled through a resin bath, further consolidated in a heated die. It allows production with a high degree of automation and lower production cost [60,61,62].

### 6.2. Advanced Manufacturing Processes

An electrostatic fibre fabrication technique called electrospinning uses electrical forces to produce continuous fibres of two nanometers to several micrometers. It serves improved physical and mechanical properties, adaptability over measure boundaries, high surface region to volume proportion, and high porosity; this way, it discovers potential in different fields of biomedical applications like injury mending, tissue designing platforms, drug conveyance, as a layer in biosensors, immobilization of catalysts, beautifiers [63,64]. The electrospinning process and additive manufacturing (AM) provides a high level of geometrical complication for the fabrication of wholly customized objects. It takes the benefit of computer-aided designing and removes mould requirements, which saves the manufacturing process’s cost and time [65,66].

### 6.3. Automated Manufacturing Techniques

Filament winding helps create axisymmetric and some non-axisymmetric composite components, such as pipe bends [67]. Powered by several pulleys, endless prepreg sheets, rovings, and yarns are made to go through a resin bath and accumulated over a spinning mandrel. Applying adequate layers on the mandrel, which has the desired form of the product, it is cured at room temperature [68,69].

## 7. Physical and Mechanical Properties of Natural Fibre Composites

Physical and mechanical properties of natural fibre composite’s (NFC’s) such as tensile, flexural, impact, and water absorption are the most general tested properties. The natural fibre selection for different applications depends on the NFC’s strength [24]. The factors that influence NFC’s mechanical properties are matrix selection, polymer interface strength, dispersion, orientation, manufacturing process, and porosity [70]. The tensile properties are improved by adding fibre to the polymer matrix because they have higher values of strength and stiffness as compared to matrices. When the weight ratio increases near the optimum values (i.e., maximum fibre loading), it increases the load distribution, which is strongly bonded with matrix and resin, thus revealing better tensile strength. Higher fibre loading is required for achieving the high performance for short-reinforced polymer composite [44]. The properties of flexural strength are completely dependent on the bottom and top surfaces of the specimen. The flexural stiffness is a criterion for measuring the deformability of the sample. The material structure’s flexural stiffness depends on two essential properties: elastic modulus (stress per unit strain) and its moment of inertia around the cross-sectional geometry of the material [24]. Fracture toughness is the most important characteristic property of the material. Impact strength is the energy required to cause damage and the failure progress in the composite. The composite toughness relies on natural stress and strain behaviour. Fibres with superior mechanical properties show the highest impact strength. Water absorption behaviour shows the hydrophilicity of the fibre to take maximum water in the presence of holes and voids. Some challenges are still present in leaf fibre-reinforced polymer composite. At first, eco-friendly fibre surface treatment should be developed and implemented. Secondly, natural fibre’s basic characteristics should be identified before it will introduce in polymer, enhancing the quality of fibre matrix adhesion. The current challenge is to make them cost-effective. The efforts to produce economically attractive composite components have resulted in several innovative manufacturing techniques currently being used in the composites industry. Additionally, flammability is one of very important phenomena that often limits the application of composites to a given area. However, very little information is available on their fire performance. Thermal stability of natural fibers, moisture content of the fibers and biodegradation and photo degradation of natural fibers is one big issue in the natural fiber polymer composite.

Different chemical treatments can resolve this problem, like alkali treatment such as NaOH. However, silane treatment plays a crucial role in leaf fibre polymer composite to enhance NFC’s physical and mechanical properties. For example, surface modification helps to improve natural fibre network structure. For example, alkaline treatment (NaOH) creates a disturbance of hydrogen bonds in the fibre, therefore increasing the fibre’s surface roughness. The alkaline treatment eliminates the assured quantity of oil, wax, inorganic salt, and lignin, which covered the fibre outer surface, uncover the short length crystallites, and cellulosic component is depolymerized. On the other hand, silane was used as a coupling agent. Silane coupling agents can decrease the number of non-cellulosic components in the fibre–matrix interface. Additionally, the silane coupling-treated natural fiber polymer composites are thermally stable as compared to other surface modifications. Leaf-extracted fibre has low density, high specific strength, and modulus. Leaf fibre, i.e., Sisal, abaca, pineapple, agave, and banana fibre used in a polymer composite, is useful in a lightweight material potential resource like packaging industry, interior paneling in the automobile industry, and house holding application. Availability and cost of natural leaf fibre much low as compared to other natural fibre. Additionally, the use of eco-friendly chemical treatment can improve the thermal stability of natural leaf fibres-reinforced polymer composite.

### 7.1. Sisal Fiber Composite

**Figure 8:**
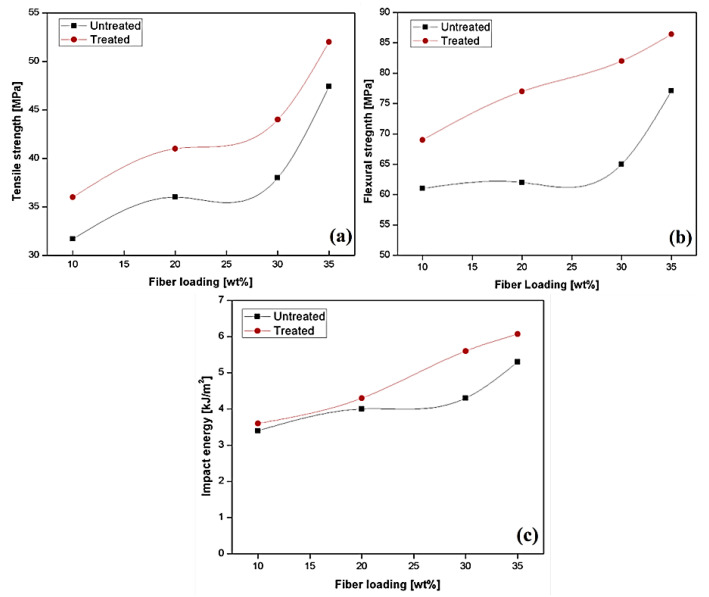
(**a**) Tensile (**b**) Flexural, and (**c**) Impact strength at fiber loading (10, 20, 30, and 35 wt. %) of untreated and 5 wt. % of alkali (NaOH)-treated sisal-reinforced epoxy composite. Adapted with the permission from Reference [72]. Copyright 2017 John Wiley and Sons.

A study on unidirectional Sisal-epoxy composite with alkali treatment of 2% sodium hydroxide (NaOH) was reported by Rong et al. [71]. The tensile property of the composite material was increased as fibre loading increased. However, a further increase of fibre- loading in matrix resulted in a decrease in the tensile strength due to the cavities present between the fiber-matrix contacts. An alkali treatment linearly increased the tensile strength because of better fibre adhesion with the matrix. The surface modification (alkali treatment) made the surface much coarser compared to untreated/epoxy composite. The flexural strength of the unidirectional sisal-reinforced epoxy composite was controlled by resistance to inter-laminar failure. It was reported that the flexural strength increased with alkali treatment. The main reason for the increase in the strength was due to epoxy, which was capable of filling up the apparent flaws resulting in better load sharing [71]. Nimanpure et al. [72] studied the 5 wt. % of alkaline (NaOH)-treated sisal fibrils epoxy composite with fibre loading 10–35 wt. %. The tensile strength of treated (NaOH) sisal composite was higher than untreated sisal composites, and as the fibre loading increased, the tensile strength increased for both treated and untreated composites, as shown in Figure 8. NaOH treatment caused the removal of the hemicellulose and lignin components. Decreasing the hemicellulose content led to the reduction of rigidity and density of the interfibrillar region. Therefore, fibrils were highly capable of reorganizing themselves along with the tensile deformation direction. Reorganization led to an increase in the transfer of load capacity among the fibril, hence increased the higher stress-bearing capacity. Lignin removal caused the elimination of micro-voids; hence the middle lamella of the fibrils was more homogeneous and plastic. Flexural strength and impact energy showed similar trends as tensile property as shown in Figure 8 [72]. The treated composite also showed good interfacial bonding between fiber and matrix.

Mechanical properties of short sisal epoxy composite with varying length 5–20 mm was reported by Maya et al. [73]. The 5 mm sisal-based composite had the least tensile strength due to short epoxy composite underwent cluster formation and not mixed uniformly with the matrix. The tensile strength initially decreased with the dispersion of shorts, but with the increase in the length of sisal fibres, the tensile strength was slightly enhanced, i.e., 32.2 MPa to 45.45 MPa. At the same time, the flexural strength and impact strength was increased with an increase in fibre length. The result indicated that with long length, the interfacial bonding of fibre and matrix was enhanced. The 20 mm length of sisal epoxy composite showed the highest mechanical properties [73]. Yan et al. [74] studied the mechanical properties of sisal lumen-reinforced epoxy composites. The epoxy resin ratio filled in the sisal lumen was adjusted by injecting in an aligned direction at the required pressure. At low injection pressure, the resin amount penetrated more in the sisal lumen and strengthened the sisal. The crack phenomenon of the resin penetrated sisal was hindered, and thus the crack propagation was decreased. Additionally, resistance towards the water uptake was increased due to the lumen space’s occupation by the hydrophobic epoxy resins. Thus, mechanical properties like tensile, flexural, impact strength and water absorption were increased with more lumen filled with the resin [74]. Beetle et al. [75] studied the fracture mechanism for randomly chopped sisal fibre reinforced epoxy composite with different loading, i.e., 15, 25, 30, 35, and 40 wt. %. The result showed that as the loading increased, the fracture toughness properties of sisal/epoxy composite were increased till 30 wt. %, but after 30 wt. %, the fracture toughness was decreased. This showed that 30 wt. % of fibre loading was the optimum value for sisal-epoxy composite. Decreasing the toughness after 30 wt. % revealed the poor interfacial bond between matrix and chopped sisal [75]. The mechanical properties of unidirectional sisal/epoxy composites prepared by resin transfer moulding (RTM) were investigated by Oksman et al. [76]. The studies explained that the tensile strength increased as the fibre loading was increased. The mechanical test results indicated that the mechanical properties of sisal-epoxy composite showed higher modulus (40 GPa) compared to the mechanical sisal test (24 GPa), i.e., absence of matrix. On the other hand, the effective strength of technical composite strength was lower than measured technical strength. This showed that the low strength weight fraction of the mechanical commanded the composite. Additionally, the more uniform sisal distribution in composite improved the fibre matrix adhesion in sisal–epoxy composites [76].

**Figure 9:**
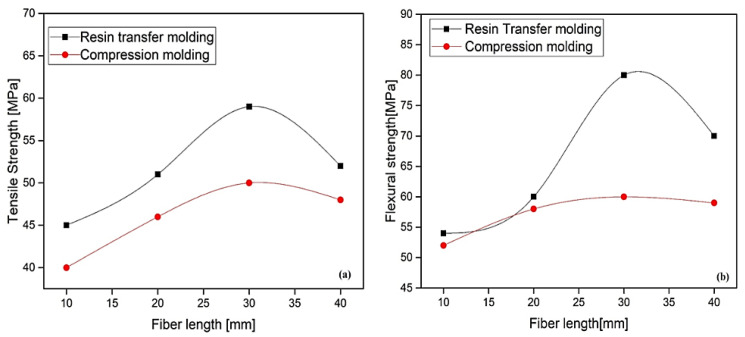
(**a**) Tensile strength, and (**b**) Flexural strength at different lengths (10, 20, 30, and 40 mm) at 30 vol. % of sisal/polyester composites. Adapted with the permission from Reference [78]. Copyright 2009 Elsevier.

Sreekumar et al. [77] reported the influence of surface modification on the mechanical and water absorption properties of sisal/polyester composites manufactured by resin transfer moulding (RTM). Chopped sisal was treated with different surface modification chemicals, i.e., permanganate, silane, benzoyl chloride, sodium hydroxide (NaOH). The chemical treatments and the physical treatment like heating (100 °C) improved the interfacial bond between fibre and polyester resin. The treatment also indicated that impact properties were decreased as compared to untreated sisal/polyester composite. The study showed that water uptake was also decreased with treatment which supported the higher sisal–polyester interaction. Properties like sorption, diffusion and permeability coefficient decreased after different treatments [77]. Mechanical properties of sisal-reinforced polyester composites fabricated by RTM and compression moulding (CM) were studied and compared. The tensile strength at different lengths for RTM and CM is shown in Figure 9a. Flexural strength at different loading for both RTM and CM is shown in Figure 9b. The mechanical properties like tensile and flexural strength were studied as a function of length (10, 20, 30, and 40 mm) and fibre loading for both fabrication methods, i.e., RTM and CM. The tensile and flexural strength of length 30 mm demonstrated the maximum strength by the RTM technique compared to CM. This maximum tensile strength and flexural strength delivered with 30 mm length at 30 vol. % of fibre loading attributed to CM due to superior interfacial bonding between fiber and matrix [78].

**Figure 10:**
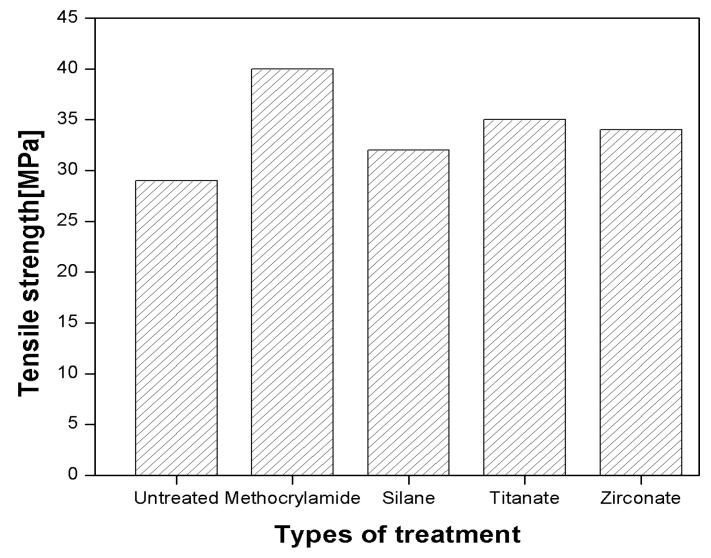
Influence of different chemical treatment on tensile strength of sisal/polyester composite (50 vol. % of sisal fiber). Reprinted with the permission from Reference [79]. Copyright 2004 John Wiley and Sons.

Singh et al. [79] reported the effect of different chemical treatments, i.e., titanate, zirconate, silane, and N-substituted meth-acrylamide, on the mechanical properties of sisal fibre-reinforced polyester composites. An improvement in mechanical properties was observed when sisal fibres were modified with surface treatments. Under dry conditions, i.e., unmodified, a decrease of 30 to 44% in tensile strength and 50 to 70% in flexural strength was reported due to higher hydrophilicity. It was observed that the extent of moisture/water absorption of surface-treated fibres had been reduced significantly by providing hydrophobicity to the surface via long-chain hydrocarbon attachment. The different surface modifications of sisal fibre showed an improvement of 15 to 33% in tensile strength and 21 to 29% in both flexural strength and modulus (silane-treated samples showed an improvement of 62% in flexural properties). This improvement indicated an improved fibre-matrix adhesion due to surface modification. The tensile strength of different treatments is shown in Figure 10 [79].

Sangthong et al. [80] studied the mechanical properties of untreated and treated sisal polyester composite. Sisal surfaces were modified by 6% NaOH, and further, the fibres were coated by ad-micellar polymerization with a poly methyl methacrylate. It was observed that both tensile and flexural properties were increased with increasing fibre loading, up to 30 vol. %, and after that, the tensile properties were slightly decreased at 40 vol. % fibre loading for all types of sisal fibres composite due to poor adhesion of fibre and matrices. Additionally, both impact and hardness properties were increased with increasing the fibre loading and length. Higher amounts of the coating were also found to impart higher impact and hardness properties. After treatment, moisture absorption of the composite was reduced to almost 50% (7.98% to 4.48%). Similarly, as the length increased from 10–40 mm at 30 vol. % loading of fibre, the tensile and flexural strength delivered maximum value at 30 mm length. At high loading, it was more difficult for the resin to penetrate the decreasing spaces between the fibres, thus causing poor wetting, and hence, a reduction in the stress-transfer efficiency between fibre-resin interface. This study exhibited the optimum value of fibre loading is 30 vol. % and 30 mm of length for all sisal-polyester composites [80].

**Figure 11:**
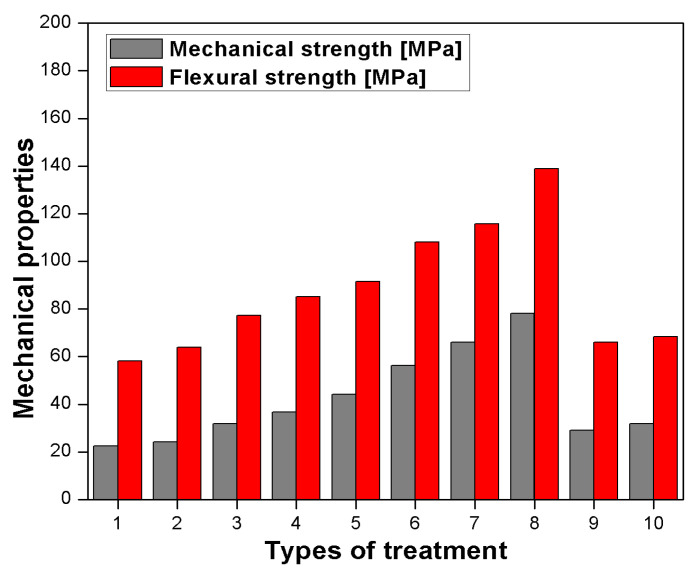
Mechanical properties of (1: Neat polyester; and different concentration of chemical treatment 2: untreated Sisal fibre; 3: 5% NaOH-treated; 4: 5% NaOH boiled; 5: 5% NaOH-treated; 6: 10% NaOH boiled; 7: 18% NaOH-treated; 8: 18% NaOH boiled; 9: 20% acetic acid-treated 10: methanol-treated) sisal/polyester composite.

The mechanical properties of sisal-reinforced polyester composites with different concentration of alkali treatment (aqueous NaOH) was reported by Khanam et al. [81]. It was evident from Figure 11 [81], that the tensile and flexural strength linearly increased with NaOH concentration. The tensile and flexural strength of sisal/polyester composite were found maximum at boiled 18% aqueous NaOH solution treatment. At boiled 18% aqueous NaOH solution, all the natural and artificial impurities were removed, creating a rough surface. The rough surface led to a better/matrix adhesion, which increased sisal/polyester composite’s mechanical properties. Influence of surface modification on mechanical properties untreated and treated sisal fibre composites. The impact of different treatments (5, 10 and 18% aqueous NaOH boiled and treated, acetic acid-treated, and methanol treated) of sisal fibre on the tensile and flexural strength of sisal fibre composites was enhanced at 18% NaOH boiled sisal fibres due to roughness of fibre after treatment and adhesion between fibre and matrix were improved. This is because alkali treatment can remove natural and artificial impurities and produce a rough surface topography. This topography offers better fibre matrix interface adhesion and an increase in the mechanical properties of the composites. There is a lot of betterment in strength and modulus with 18% NaOH boiled sisal fibres, which again shows, at high temperature, a rougher surface might have taken place. This rough surface increases the interface bonding between the fibre and the matrix.

### 7.2. Abaca Fibre Composite

Liu et al. [82] studied the effect of chemical treatments on transverse tensile properties of unidirectional abaca/epoxy composite. The transverse tensile test was performed to analyze the linkages in a unidirectional abaca-epoxy composite. It was revealed that the transverse tensile behaviour of unidirectional composite purely depended on the bond between fibre and matrix. The chemical composition of lignin and hemicellulose showed a reducing effect on transverse tensile behaviour. Salinization and light mercerization (1.0 wt. % NaOH solution for 5 min) played a key role in enhancing the/epoxy interfacial strength, while a heavy surface modification, i.e., 5.0 wt. % NaOH solution for 30 min decreased the fiber-matrix adhesion, and led to resin interface modification and affected the lumen size, which weakened the interfacial bonding between fibre and matrix [82].

**Figure 12:**
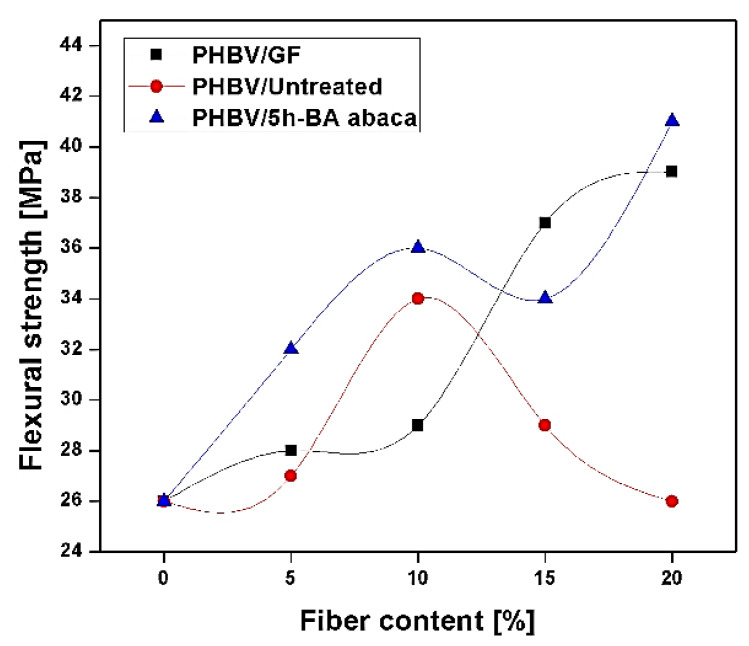
Flexural strength of biodegradable polyester composite-reinforced with glass fiber (PHBV/GF) composite, PHBV/untreated abaca composite, and PHBV/5h-BA abaca composite at different fiber loading (5–20 wt. %). Adapted with the permission from Reference [84]. Copyright 2002 John Wiley and Sons.

The influence of alkali treatment on abaca-reinforced composites was reported by Cai et al. [83]. The abaca fibres were immersed for 2 h with an aqueous solution of NaOH having a different concentration of 5, 10, and 15 wt. % for better penetration of NaOH solutions into the bundles. The untreated abaca showed a tensile strength of 717 MPa and Young’s modulus of 18.6 GPa. The abaca fibres treated with 5% of NaOH solution showed an increment in tensile strength by ~8% and in tensile modulus by 36% in comparison to untreated abaca fibres. Above 5% NaOH treatment, i.e., 10% and 15% of abaca, the mechanical strength was found to deteriorate due to excess elimination of binding material (cellulose and hemicellulose) due to interactions of highly concentrated alkali environment. The 10 and 15% NaOH-treated abaca fibres-reinforced epoxy composites maintained their tensile strength of 682 ± 83 MPa and 670 ± 26 MPa, respectively, but their Young modulus was decreased by 34% and 49%, respectively. Abaca bundles treated in 5 wt. % NaOH showed high cellulose crystallinity and minimal fibrillation and exhibited tremendous interfacial adhesion with epoxy resin. Results indicated that the low concentration of alkali treatments (5%) was beneficial in improving the surface properties and its performance of abaca fibres for leading composites applications [83]. Shibata et al. [84] studied flexural strength of untreated abaca fiber, and butyric anhydride (BA)-treated abaca fiber-reinforced with biodegradable polyester, i.e., poly (3-hydroxybutyrate-co-3-hydroxyvarelate) (PHBV) composite, and compared with PHBV-reinforced with GF, i.e., PHBV-GF composites. The abaca fiber was treated with butyric anhydride (BA) for 5 h and reinforced with PHBV biodegradable polyester, represented as PHBV/5h-BA abaca in Figure 12. The result showed that PHBV/5h-BA abaca-treated composite showed maximum flexural strength as compared to untreated PHBV/abaca composite (PHBV/untreated). On the other hand, when fiber loading increases, i.e., 5–20 wt. %, the flexural strength at 20 wt. % of PHBV-treated abaca showed the highest flexural strength (41 MPa) as compared to PHBV-untreated abaca fiber and PHBV-GF composite. This showed that the interfacial adhesion between fiber and matrix became superior due to treatment, as shown in Figure 12 [84].

**Figure 13:**
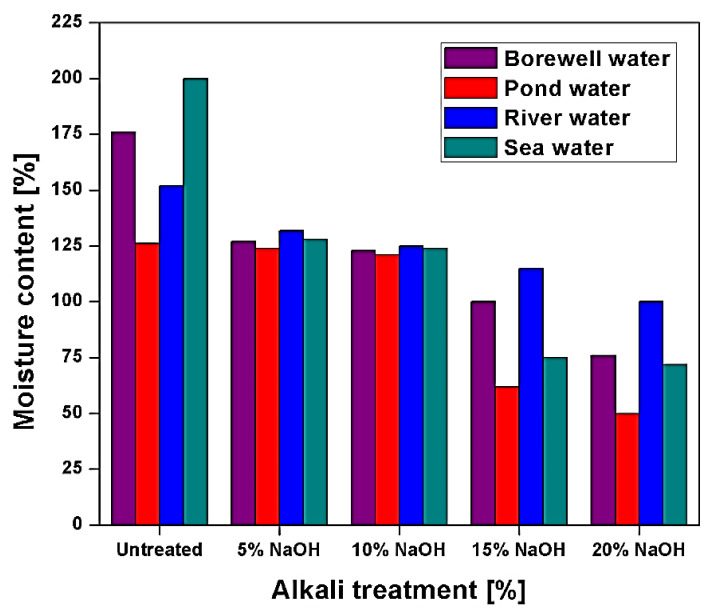
Water absorption tendency of untreated and alkali (NaOH)-treated single abaca fibre from different water sources [85].

Punyamurthy et al. [85] investigate the water absorption behaviour of untreated and alkali-treated abaca fibre with different water resources. Moisture absorption studies are done with four different water sources, i.e., seawater, pond water, river water, and borewell water. Alkali treatment of abaca fibre is done by 5–20% of NaOH. Result suggests that as the concentration of treatment increases, the water absorption of abaca fibre is decreased, as shown in Figure 13 [85]. Pond water showed the least water absorption as the concentration of the alkali treatment is increasing; this is due to excessive removal of lignin and hemicellulose content which reduces the hydrophilicity of the abaca fibre [85].

**Figure 14:**
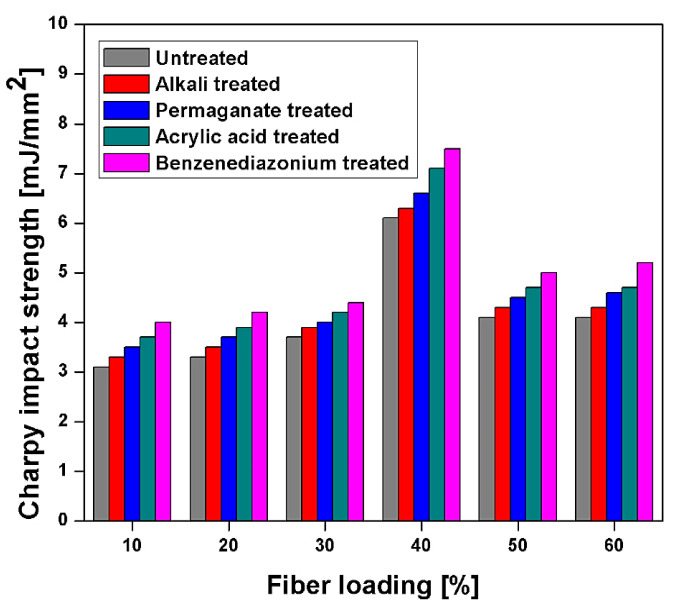
Influence of chemical treatment on impact strengths at different fiber loading (10–60 wt.%) of abaca-reinforced epoxy composite.

Punyamurthy et al. [86] studied the Charpy impact strength of different surface modification techniques for abaca-reinforced epoxy composite. It is treated with different chemical treatments, i.e., Alkali (6% NaOH), Acrylic acid (1%), Permanganate treatment (0.5% KMnO_4_), and benzene-diazonium chloride solution for better surface modification. The study is done with different loading 10, 20, 30, 40, 50, and 60 wt. %. Further, 40 wt. % loading of all the different treatments shows maximum impact strength as shown in Figure 14 [86]. This is because at 40% loading, composite showed better distribution in the matrix, low fractures, and better transfer of load from fibre to the matrix. After 40% of loading, impact strength decreased due to poor bond and less transfer of load from the matrix to fibres. Untreated composites showed poor mechanical properties due to easy pullout from the interfacial between the fibre and matrix. Simultaneously, alkali treatment removes non-cellulosic components and more hydroxyl (-OH) groups in the surface to improve fibre-matrix adhesion. KMnO_4_ treatment deliver better interlink at the interface by accomplishing rough surface to improve the fiber-matrix bond. Similarly, acrylic acid improves stress transfer capability to the fibres and matrix to enhance the impact strength.

Paglicawan et al. [87] investigated the mechanical properties of plasma and alkali-treated abaca/epoxy polymer composite. The abaca fibre is put between the electrode plate in which plasma polymerization takes place. Acrylic acid was used as a monomer for surface modification by plasma polymerization. On the other hand, hybrid treatment of 2% NaOH (alkali-treated) was used for abaca fibre at a different times for plasma treatment. The tensile strength of the composites treated with plasma was better for those with hybrid treatment and no treatment due to better adhesion. The composite’s water absorption is shown to reduce with increase plasma disclosure time and reduced with combined treatment of NaOH and plasma [87]. Chemical treatment of waste abaca-reinforced fly ash is investigated by Malenab et al. [88]. Alkali pre-treatment of 6% NaOH is introduced to remove impurities result create a rough surface. NaOH-treated abaca is then treated with aluminum sulphate Al_2_(SO_4_)_3_. Chemical treatment of waste abaca enhances the structure and chemical composition, and high tensile strength is achieved by removing non-cellulosic components like pectic, wax, hemicellulose, and lignin. Geopolymer itself is coated in the composite to become a barrier to the pre-treated composite’s thermal degradation. The aluminum sulphate treatment indicates that it deposits the Al(OH)_3_ and roughens the surface for better fibre-matrix adhesion to protect the fibre from the harsh environment [88].

Ahmed et al. [89] reported the effect of silane-modified and vinyl ester on the mechanical properties of abaca composites. A vinyl ester with 1 wt. % of Hexamethyldisiloxane (HMDS) silane modification with dispersion time of 120 min showed 10% more tensile strength than untreated vinyl ester composite due to the homogeneity of the dispersion of resin components after stirring for an appropriate time. On the other hand, at 2 wt. % of HMDS silane modification-vinyl ester abaca fibre, the tensile strength increases about 18% because vinyl ester is adsorbed to the hydroxyl (-OH) groups of natural abaca fibres by hydrogen bonds on the surfaces and improve adhesion between fibre and matrix. Similarly, 2 wt. % of HMDS-vinyl ester composite showed higher flexural strength. Water absorption is at least at 3 wt. % of HMDS-vinyl ester composite due to the removal of non-cellulosic components and decreases the composite’s hydrophilicity [89].

Liu et al. [90] studied alkali (NaOH)-treated abaca-reinforced friction composites. The chopped abaca of 10 mm length and treated with 3 wt. % of alkali treatment and then soaked with 1 wt. % of sulphuric acid (H_2_SO_4_) solution. The tensile strength of treated abaca is increased by 5.19%, and elastic modulus is enhanced by 2.61% MPa. The result illustrates that the stiffness and brittleness of the abaca composite improved after alkali treatment. The impact strength of specimens is improved initially and then decreased with the increase of abaca fiber loading. At 3 wt. % of abaca fibre shows maximum impact strength because of better interfacial bonding between matrix and fibre due to chemical treatment [90]. Batara et al. [91] studied the influence of chemical treatment of alkaline and permanganate treatment on abaca’s mechanical properties. The mechanical properties illustrate the combination and individual of both the chemical treatment. It is treated with 10% of NaOH with different concentrations of permanganate treatment, it is also exposed individually for permanganate for a respective time. The result indicates that the tensile strength exposed to 0.125% potassium permanganate for 3 min showed maximum tensile strength. This is due to treated fibers having fewer hemicellulose and lignin as compared to the untreated, as treatment is known to eliminate the lignin and hemicellulose component. Those treated directly with KMnO_4_ exhibited the cleanest surface which enhances the mechanical property [91].

### 7.3. Pineapple Fibre Composite

**Figure 15:**
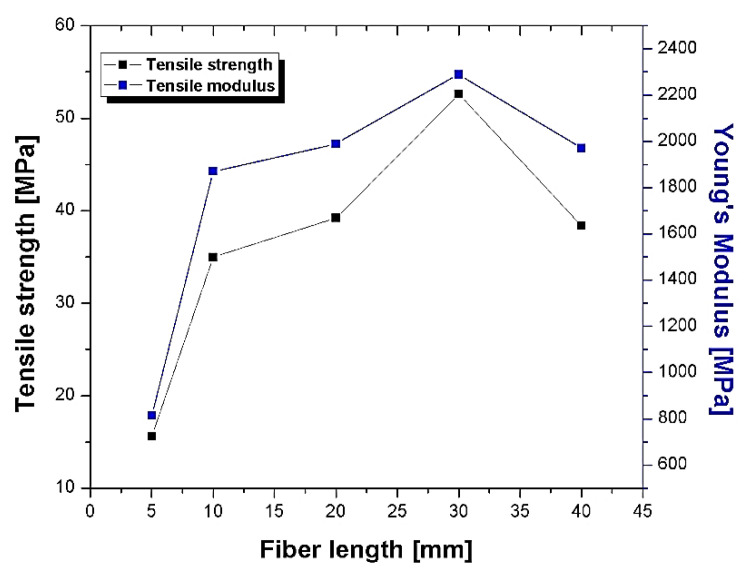
Tensile properties of chopped pineapple leaf fiber (PALF) with different fibre lengths (5–40 mm) at 30 wt. % of PALF reinforced with polyester composite. Adapted with the permission from Reference [92]. Copyright 1998 John Wiley and Sons.

The influence of different chemical treatments (alkali, silane A-172) on fibre length and loading on mechanical properties of different chemical-treated pineapple-reinforced polyester composites was reported by Devi et al. [92]. The chopped ones with different lengths 5–40 mm were studied, and results indicate that after the length is increased from 5 mm to 30 mm, it shows a decreasing trend. At 30 mm long fibre, the tensile, flexural, and impact strength is the maximum due to entanglements that occur above an optimum size. Similarly, as fibre loading increases, the tensile and flexural strength is increased at 30 wt. %. This showed that 30 mm and 30 wt. % is the optimum value of pineapple polyester composite in this investigation, as shown in Figure 15 [92]. At different nature of treatment silane, A-172-treated showed superior mechanical properties as compared to alkali, and other silane-graded-treated composites.

Mishra et al. [93] reported mechanical properties of different surface modifications on pineapple leaf fibre (PALF) polyester composite. Detergent washed (ethanol and benzene) alkali-treated (5%, NaOH), grafted acrylonitrile (AN), and cyanoethylated PALF polyester composites were fabricated and compared. The tensile strength of the alkali-treated pineapple polyester composite showed a maximum value of 44.77 MPa because alkali treatment removes the impurities from the surface of the material and enhances the fibre-matrix adhesion as compared to other surface modifications. Additionally, the grafted AN modification at 10 wt. % concentration the flexural strength is maximum due to wettability and better fiber-matrix adhesion. However, a further increase in grafting tends to decrease in flexural strength by 20% drastically due to significant breakage and delamination [93]. Water absorption and mechanical properties of the pineapple-reinforced polyester composite are reported by Devi et al. [94]. PALF polyester composite showed a 123% increment in the water absorption capacity as loading increased from 30 wt. % to 40%, due to the presence of hydroxyl groups which enhance the absorption of water in composite by developing hydrogen bond. Tensile strength of the composite increases with an increase in fibre loading. At 40 wt. % of loading, maximum tensile strength of 63.3 MPa is shown because of the better interface between fibre and matrix [94].

**Figure 16:**
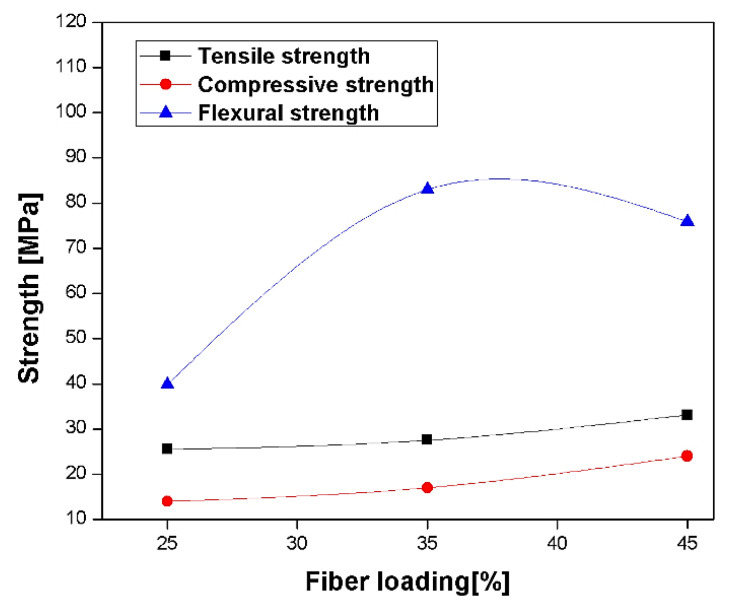
Tensile, Compressive, and Flexural strength of pineapple-reinforced polyester composite at different fiber loading (25, 35, and 45 wt. %). Adapted with permission from Reference [95]. Copyright 2019, with permission from Elsevier.

**Figure 17:**
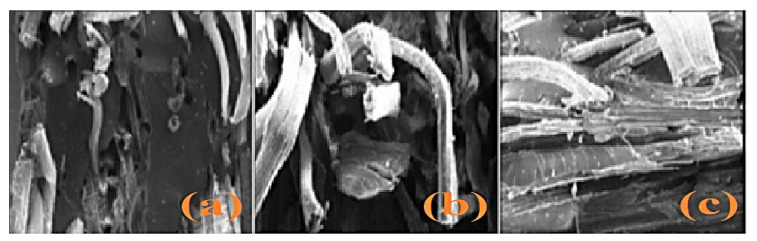
(**a**–**c**) SEM images of the flexural fractured specimen at different fiber loading, (**a**) 25 wt. %, (**b**) 35 wt. %, and (**c**) 45 wt. % of pineapple-reinforced polyester composites. Reprinted with the permission from Reference [95]. Copyright 2019 Elsevier.

Mechanical and morphology study of PALF reinforced in unsaturated isophthalic polyester composite was reported by Senthil Kumar et al. [95]. Figure 16, showed that the tensile and compressive strength is increased as the fibre loading increases from 25% to 45 wt. %. The improved strength of composites with high fibre loading is due to the improved interfacial adhesion. At the same time, flexural strength is maximum at 35 wt. % of loading. The decrement of flexural strength at 45 wt. % loading, due to undistributed applied stress transfer between the fibre and matrix at higher interaction within the matrix and poor dispersion. In Figure 17a–c [95], the morphology study of the composite in the flexural fracture test is presented. The properties showed a strength of 25 wt. % composite is low, adhesion between fibre and the matrix is improved. On the other hand, it was bending and the poor dispersion of shorts fibre which did not allow the appropriate load transfer. Further, 45 wt. % loading shows the more significant contact between the fibres due to the reduction of the matrix, which can resist the shearing movement across the fibres, and it manages part of the decrement in flexural strength [95]. Impact strength of 3 mm chopped PALF polyester composite with chemical treatment, i.e., sodium hydroxide (NaOH) and potassium hydroxide (KOH) with loading 25%, 35%, and 45 wt. % was observed by Senthil Kumar [96]. The impact strength at 25 wt. % loading composites with NaOH-treated has maximum impact strength (70 J/m) due to better interfacial bonding between the fibre and matrix and removal of soluble oily contents from the fibre as compared to untreated and KOH-treated [96].

**Figure 18:**
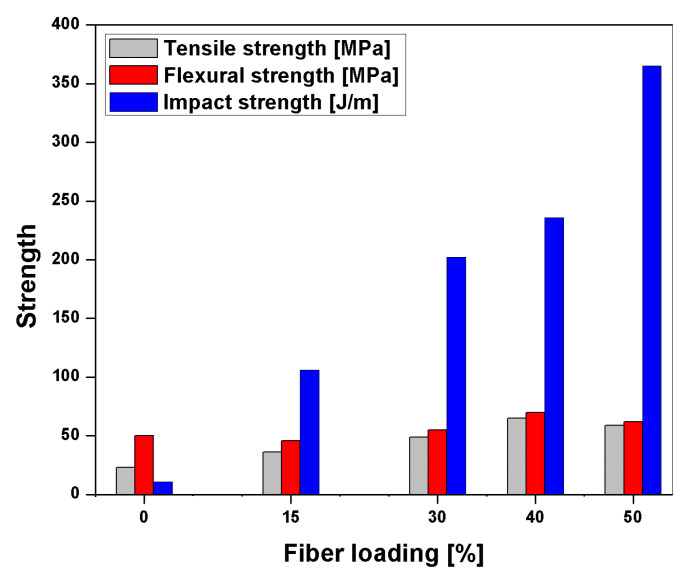
Mechanical properties at different fiber loading (0–50 wt. %) of pineapple leaf fiber-reinforced polyester composite. Adapted with the permission from Reference [98]. Copyright 2011 John Wiley and Sons.

Krishnasamy et al. [97] studied the calcium hydro-oxide (Ca(OH)_2_)-treated PALF/polyester composite. The chopped 3 mm PALF is treated with 7% of calcium hydro-oxide at different loading, i.e., 25%, 35%, and 45 wt. %. From this investigation, the damping properties are improved due to effectiveness in the load distribution from fibre to the matrix. While maximum impact strength (59.27 J/m) was obtained for 35 wt. %-loaded composite with treated fibres for strong interface bonding. The result showed that PALF is suitable for applications requiring vibration damping and impact resistance [97]. Devi et al. [98] studied PALF reinforced in polyester composite’s mechanical properties with different loading at 30 mm length with 600 aspect ratio (length to diameter). The loading of PALF, i.e., 0, 15, 30, 40, and 50 wt. %, the tensile and flexural strength increases linearly from 0–40 wt. %. Moreover, 40 wt. % of PALF showed maximum tensile and flexural strength due to better adhesion of fibre/matrix. After 40 wt. %, a decreasing effect was shown due to poor dispersion, while impact strength showed an increasing effect as the content was increased. The impact properties of composites increase with the fibre microfibrillar angle when it reaches a maximum value at 15°–20°. In this investigation, the microfibrillar angle is 16°, so the PALF-polyester composite showed superior impact strength, as shown in Figure 18 [98].

The performance of surface treatment on a single PALFepoxy composite was studied by Payae et al. [99]. The PALF is treated with 5% of NaOH for surface modification. Epoxy resin in toluene (1 wt. %) was coated in PALF. The untreated showed the least interfacial shear strength (26.68 MPa) due to poor interlocking, while the alkaline treated showed higher strength (52.39 MPa) compared to the untreated due to increment in surface roughness and area, and thus build up the interaction between pineapple fibre and epoxy. On the other hand, epoxy-coated composite showed a relatively higher interfacial shear strength of fibre about 66.46 MPa; this is due to better load sharing and high-stress reinforcement in the single PALF reinforced-epoxy composite. Similarly, tensile strength showed a similar pattern as interfacial shear stress due to improved stress transfer efficiency at the interface of the matrix in the alkali-treated composite as compared to epoxy-coated composite, as shown in Table 4.

Lopattananon et al. [100] reported the interfacial adhesion and mechanical properties by surface modification of PALFreinforced in epoxy composites. The PALF is treated with 5% NaOH, 1% di-glycidyl ether of bisphenol A (DGEBA), and 5% NaOH with 1% DGEBA. The flexural strength of 5% NaOH and 5% NaOH with 1% DGEBA showed maximum strength of about 156 and 155 MPa, respectively. Flexural strength is efficient due to fiber-matrix adhesion and more efficient stress transfer. Impact strength of 5% NaOH with 1% DGEBA-treated had superior strength, which means the better combined synergetic effect of NaOH/DGEBA with combined adhesion and fibre toughness. On the other hand, interfacial shear strength of NaOH/DGEBA surface modification showed maximum strength, which suggests that the grafting site of a hydroxyl group (-OH) of lignocellulosic is more efficient as compared to other treatments. Longitudinal tensile strength of natural rubber (NR) composites were at a different percentage of NaOH-treated PALF. The PALF treated with different concentrations of alkali, i.e., 0, 1, 2, 3, 5, and 7%, showed maximum tensile strength as 10.02 MPa treated with 5% NaOH. This indicates that at 5%-treated, PALF showed the effective surface area available for bonding with the matrix and chemical constituents like lignin and hemicellulose from a multicellular as compared to untreated and other percentages of treatment [101]. Mechanical properties of the jute–pineapple–glass in different resin, i.e., epoxy and polyester composite, are studied by Reddy et al. [102]. Volume fraction of hybrid is varied from 0.18, 0.24, 0.30, 0.36 and 0.42% respectively. Epoxy resin composite showed higher strength as compared to polyester resin due to better stress transfer in epoxy matrix resin. At 0.42% of volume fraction of both polyester and epoxy composite, maximum tensile, flexural, and impact strength is shown, but epoxy-fabricated composite showed higher strength as compared to polyester composite. Other thermoset resins like phenol-formaldehyde (PF) and vinyl ester also play a key role in the performance of the natural composite’s mechanical properties. Mangal et al. [103] reported the thermal conductivity of pineapple leaf fibre/phenol formaldehyde composite with different weight fractions of PALF, i.e., 15, 20, 30, 40, and 50%. PALF/PF composites thermal conductivity showed a decreasing trend with increasing PALF fibre content varying from 15 to 50 wt. %. Phenol formaldehyde composite could not improve thermal conductivity because PF is not provided a conductive path to the heat energy to the PALF/PF composite material [103]. Pre-treatment of PALF/vinyl ester composite on tensile properties of the composite is studied by Mohamed et al. [104]. Sodium hypochlorite (NaOCl) solution is used to improve PALF composite to enhance the mechanical properties. Tensile strength and modulus with bleaching effect of NaOCl help to improve about 123% and 35% respectively. This study also suggests that an increase in bleaching reduces the strength due to excessive removal of a cellulosic component. However, fine PALF strands are higher as compared to untreated PALF bundles in terms of tensile strength by 155.3% and modulus by 134.3%, due to better adhesion [104]. PALF fabricated with vinyl ester resin with different loading was investigated by Mohamad et al. [105]. In this investigation, liquid compression moulding (LCM) is used to enhance PALF-reinforced vinyl ester composite properties, mostly water absorption. Cost-efficient pretreatment using a dilute aqueous sodium hypochlorite solution (NaOCl) is used to treat PALF, resulting in enhanced mechanical properties and reduced water absorption. So, using PALF bleached with a higher concentration aqueous NaOCl solution did not improved composite thermal stability because it does not provide a conducive way for heat energy to reach the PALF/PF composite material. This study indicates that aqueous NaOCl may be beneficial in producing vinyl ester composites with improved thermal stability [105]. In conclusion, pineapple leaf surface modification with the different matrix can be cost-effective and add value to composite materials.

### 7.4. Banana Fibre Composite

**Figure 19:**
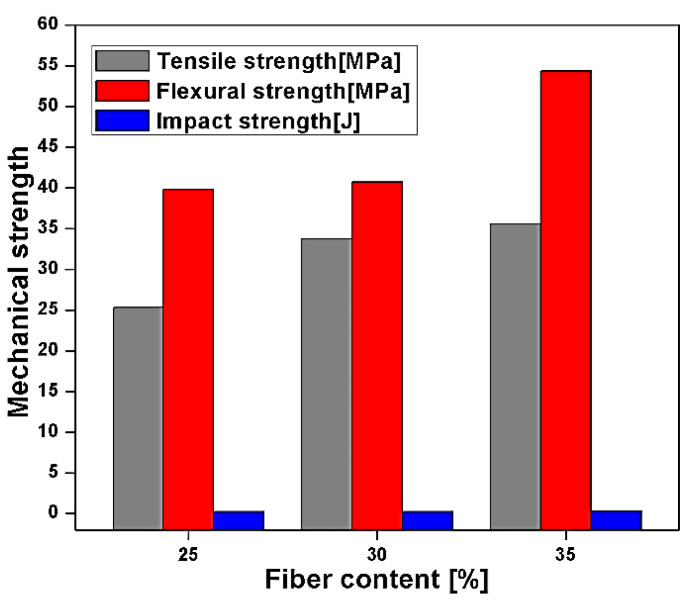
Mechanical strength of chopped fiber length (30 mm) at different fiber loading (25, 30, and 35 wt. %) of banana-reinforced epoxy composites [106].

Short banana leaf, micro, and macro banana particle reinforced epoxy composites are studied by Jayaseelan et al. [106], with different fibre loading, 25, 30, and 35 wt. % respectively. The short banana is chopped into 30 mm length and reinforced in epoxy resin without treatment. The result illustrates the tensile, flexural, and impact strength of short banana fibre at 35 wt. % is a maximum of about 46.1% and 63.48% times higher than micro and macro banana particles. The tensile and flexural strength of the short banana fibre is higher due to higher compatibility with epoxy resin and higher load-bearing capacity, as shown in Figure 19 [106]. Impact strength of short and macro banana epoxy resin showed a similar result, but microparticle of banana showed decreasing trend due to insufficient interlocking and breakage of the specimen with initial crack propagation [106].

Prasad et al. [107] reported mechanical and water absorption behaviour of banana-reinforced epoxy composite. The banana is treated with 10% NaOH for surface modification. Banana is reinforced with three different materials: sponge gourd, cyperus pangorei, and palm. From the experimental outcome, the alkali-treated banana reinforced with sponge gourd showed the highest tensile strength (40.125 MPa), flexural strength (68 MPa) due to better stress transfer from fibre to the matrix. Additionally, impact energy and water absorption showed maximum value, i.e., 6.66 Joules and 2.85% respectively. The banana-reinforced sponge gourd composite material reduced the hydrophilicity due to chemical treatment. The compressive strength was also enhanced by 3 times, i.e., 300 MPa, compared to other banana-reinforced composites [107]. Mohan et al. [108] studied the mechanical property of untreated, treated, and nano-clay-treated banana-reinforced epoxy composite. DGEBA based epoxy resin in banana leaf with 40 mm length and 40 wt. % is used to the fabricated composite material. Untreated composite compressive strength of infused nano-clay banana composite showed maximum strength due to higher cellulosic component concentration with alkaline treatment. Tensile, flexural, and interfacial strength of the nano clay composite also showed higher values because of the approximate 15% cellulose component since cellulose is a hard, crystalline, and robust structure. Therefore, the mechanical properties are superior as compared to treated and untreated composite. The untreated composite showed low strength due to lower cellulosic components and easy pull with poor adhesion [108].

**Figure 20:**
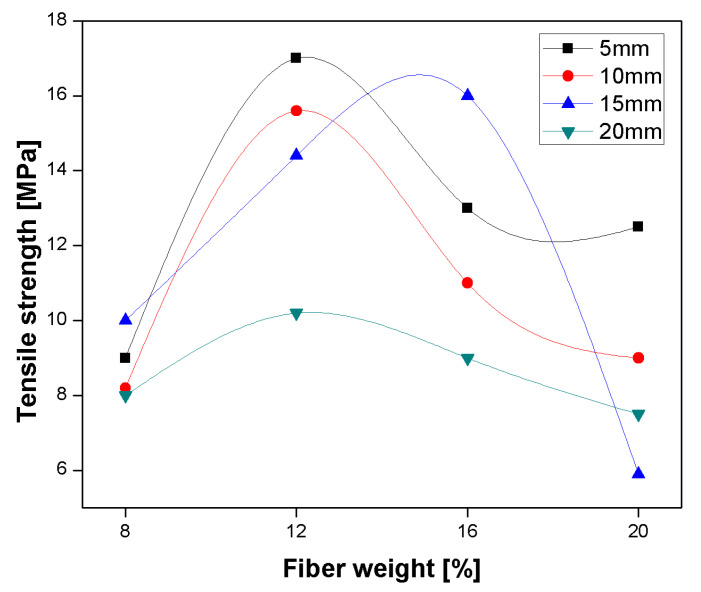
Tensile strength of chopped fibre (5 mm) length at different fibre loading (8, 12, 16, and 20 wt. %) of banana/epoxy composite.

The effect of surface modification with different alkali treatment concentrations (NaOH) on the banana-reinforced epoxy composite is reported by Venkateshwaran et al. [109]. Mechanical properties of banana/epoxy composite with different concentrations of 0.5, 1, 2, 5, 10, and 20% NaOH are studied, respectively. The result showed that 1% NaOH concentration of composite showed 52% higher tensile and 16.65% flexural strength due to NaOH treatment which converts Cellulose-I to Cellulose-II with lattice transformation as compared to untreated, and further incremental damage the fibre reduces the strength of fibres. Additionally, the impact and water absorption of treated banana/epoxy composite at 1% NaOH concentration show the superior result, with uniformly distributed stress and reduced hydrophilicity compared to untreated composite [109]. The length (5, 10, 15, and 20 mm) and loading (8, 12, 16, and 20 wt. %) of epoxy-based banana composite showed a superior effect on the composite mechanical property. Tensile strength at 5 mm and 12 wt. % loading showed maximum strength, and by further increasing the fibre content, the strength is decreased, due to poor fibre-matrix adhesion and its higher content in the matrix as shown in Figure 20. Flexural strength at 15 mm at 16 wt. % showed a maximum value of 57.53 MPa. Impact strength is increased as loading in wt. % increases. The maximum value of impact strength was obtained at 20 mm length 16 wt. %, further increment in fibre loading impact strength decreases due to the breakage of the specimen with initial crack propagation [106,110].

A woven mat is shown with three different techniques for banana fiber, i.e., plain, twill, and the basket respectively. The mechanical study reported that weave construction plays an important role inconclusive the mechanical properties of the different weave composite [111]. It also concluded that the composite made of plain weave pattern has better tensile flexural and impact strength than twill and basket types due to better weave of fibres in warp and weft direction in the plain weave as compared to twill and basket form [112].

The influence of surface treatment on fiber–matrix adhesion in the banana-reinforced polyester composite is investigated by Pothan et al. [113]. The fibre is treated with alkaline (NaOH) and different silane-treated, i.e., A151(vinyltriethoxysilane), A174 (Υ-methacryloxypropyltrimethoxysilane), A1100 (Υ-aminopropyltriethoxysilane), Si69 bis(tri-ethosilyl propyl) tetra sulphide, F 8261(1H, 1H, 2H, 2H-peruorooctyl tri-ethoxy silane). Alkali treated with 1% NaOH showed maximum tensile and flexural modulus because it increases the surface area by removing non-cellulosic components like lignin, i.e., improved packing of the cellulose chains after the lignin dissolution and removal of hemicellulose from the fibre surface. These results also suggested a larger contact area between fibre and polyester resin enhanced the composite’s tensile strength, as shown in Table 5. Silane treatment also improved the mechanical properties of the composites. Silane A174 and untreated showed maximum impact strength because silanes endured hydrolysis to form silanols, then further reacted with the hydroxyl (-OH) group cellulose. Impact strengths of alkali, silane and acetylated-treated are shown in Table 6. The organofunctional group of the treated silanes forms interpenetrating polymer networks with the polyester, which improved the mechanical strength.

**Figure 21:**
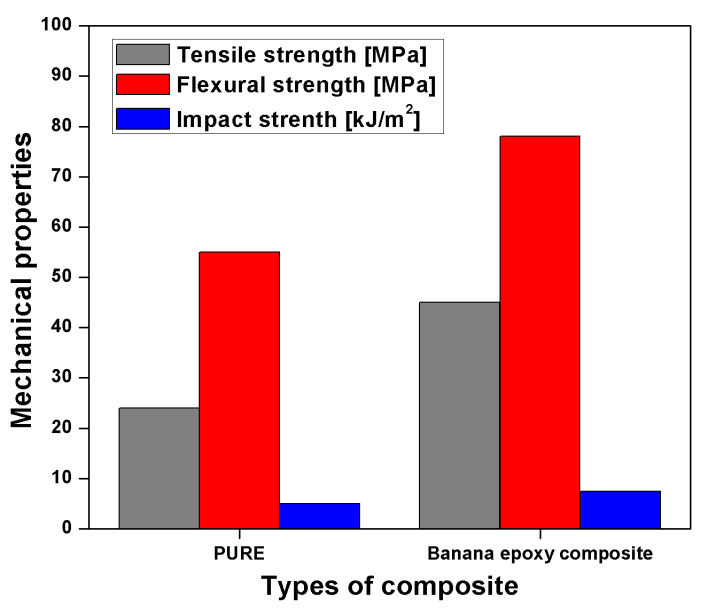
Mechanical properties of pure epoxy and pseudo-stem woven fabric banana-reinforced epoxy (BRE) composite.

Mechanical properties of pseudo-stem woven fabric banana-reinforced epoxy (BRE) are compared with pure epoxy composite investigated by Maleque et al. [114]. Tensile strength of the banana-reinforced epoxy composite is increased by 90% due to better adhesion between and matrix than pure epoxy. Flexural and impact strength also enhanced by 37.84% and 40%, respectively, due to better stress transfer between fibre and matrix, as shown in Figure 21 [114]. This investigation also suggests that the banana weaved epoxy composite showed a ductile unveiling least plastic deformation [114].

Mechanical and tribological properties of banana leaf-reinforced epoxy composite treated with Aluminium hydroxide Al(OH)_3_ are reported by Shivamurthy et al. [115]. The banana leaf fibre contents in this investigation was 5 and 10 wt. % with different Al(OH)_3_ concentrations, i.e., 2.5, 5, and 7%. The tensile strength is maximum at higher fibre content due to many fibres per unit area being reinforced with the matrix. Hardness for 5% and 10% banana-reinforced composite showed increment with the increment in the content of Al(OH)_3_ and at 5 wt. % of filler loading. However, besides filler increases, it reduces the hardness due to voids. Additionally, water absorption increased with the increase in the content of the filler in the composite. Further, an increase in the Al(OH)_3_ filler content (5 wt. % and 7.5 wt. %) in 10% banana and filler content showed a synergistic effect that resists the specific wear rate (SWR), but 10 wt. % of loading with SWR at 30 and 40 N showed the least wear as compared to 5 wt. % of a banana-filler content composite of banana and filler contribute to wear resistance. However, at higher filler content, it degrades the mechanical properties of the composite [115]. Mechanical and hydrophilicity of banana epoxy composite with the effect of graphene oxide are studied by Bharadiya et al. [116]. It is chopped into 5–7 mm length with 5 wt. % loading with different concentrations of graphene oxide, i.e., 1, 2.5, and 5 wt. %. The result showed that as the concentration of graphene oxide increases, the mechanical strength (tensile, flexural, and impact) and hydrophilicity is enhanced by 275%, 242%, 239%, 240%, and 296%, respectively, as compared with neat epoxy. This is due to an increase in the hydrophilic groups (-OH) with an increase in the Graphene Oxide (GO) concentration. A percentage of 5 wt. % concentration of GO showed maximum strength because both synergetic effect and better adhesion are higher than pure epoxy [116]. Pothan et al. [117] studied the effect of length (10, 20, 30, 40 mm) and loading, i.e., 10, 20, 30, and 40 wt. % respectively, and also silane treatment with different concentrations of banana polyester composite. The result indicates that as the fibre loading increases, the composite strength also increases due to better fibre-matrix adhesion. Lower loading acted as a flaw, and the percentage of loading is not enough to convert high strength plastic. At 20 mm, the silane-treated 0.5 wt. % concentration showed the maximum value of about 28% because the silanol group reacts with the hydroxyl group, which enhanced interlocking between the matrix. The improvement of impact strength is improved by 341% at 40 wt. % loading due to extra energy dissipation at plastic deformation [113,117].

**Figure 22:**
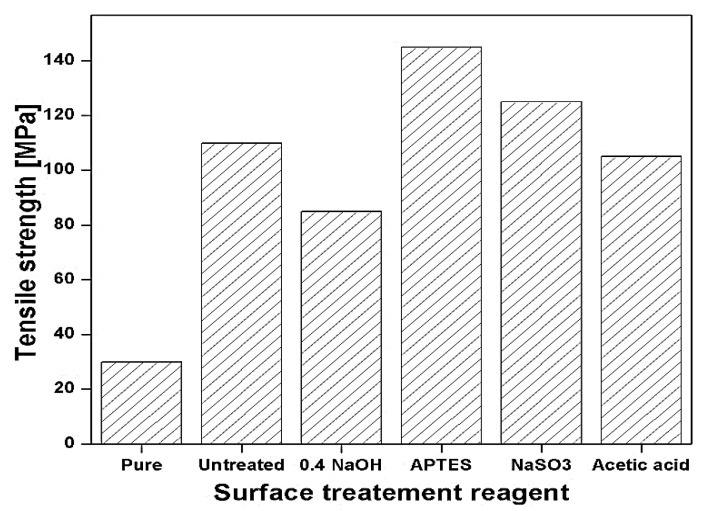
Tensile strength of neat epoxy, untreated fibre, and different surface modification reagents of the banana-reinforced polyester composite.

The influence of surface modification reagents on tensile properties of matted banana-reinforced polyester composites is reported by Ike-Eze et al. [118]. The pre and post-surface modifications of banana are done with different NaOH concentrations, i.e., 0.2 M, 0.4 M, 0.6 M, 0.8 M, and 0.10 M, respectively, for 30 min to 4 h. In this study, the pre-alkali treatment is done with NaOH, and post-alkali treatment was done by sodium sulphate (Na_2_SO_3_), 3-aminopropyl-triethoxy silane (APTES). The high tensile properties of the silane treatment fibre composites were decreased by the number of the -OH group, which means the hydrolyzable group formed by silanols which react further within the fibres form a stable covalent bond which is more adaptable with the hydrophobic (water repellent) polyester resins. At 0.2M, NaOH-treated with 1-hour soaked treatment has the maximum value because of the removal of the non-cellulosic component and higher concentration damage and excess removal of the non-cellulosic component with the cellulosic component as shown in Figure 22 [118].

**Figure 23:**
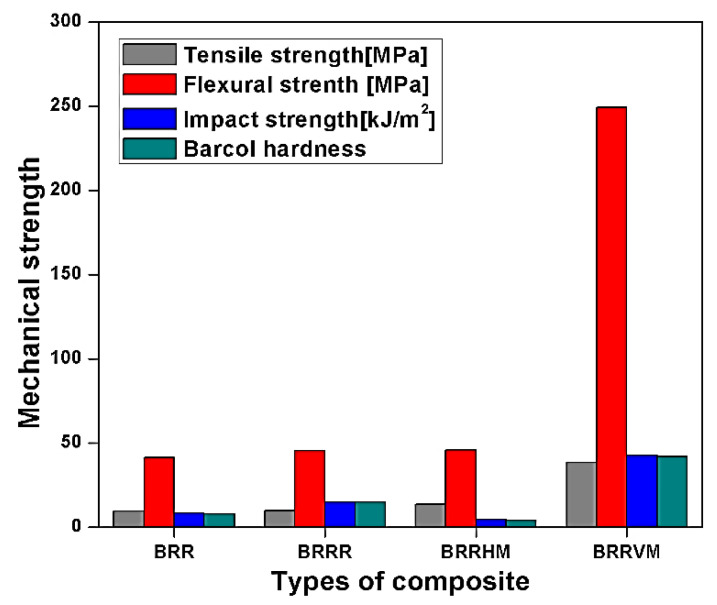
Mechanical properties of different orientation forms of banana ribbon-reinforced polyester composites.

Different forms of banana ribbon orientation, i.e., Banana Ribbon Random (BRR), Banana Ribbon Rope Random (BRRR), Banana Ribbon Rope Horizontal Mat (BRRHM), and Banana Ribbon Rope Vertical Mat (BRRVM) reinforced in polyester composites are reported by Karthikeyan et al. [119]. Tensile and flexural properties of random oriented are studied; result outcomes from the study suggest that BRRVM showed extensively high tensile and flexural strength due to resistance towards breakage under applied tensile stress. Impact strength and hardness are also superior to BRRVM because the distribution of vertical mat orientation is high as compared to other random orientations, as shown in Figure 23 [119]. At the same time, the BRRVM thermal conductivity of BRRHM and BRRVM/polyester composites decreases by 28% and 32% respectively. This performance is due to the mat form orientation of BRR [119].

Mechanical properties of Banana Woven Fabric-Reinforced (with varying wt. % 5, 10, 15, and 20) polyester composites are investigated by Mariatti et al. [120]. Flexural strength and modulus of a banana unsaturated polyester composite at 5, 10, 15, 20 wt. percentage showed similar strength but at 10 wt. % showed higher strength, i.e., 43 MPa and 1.9 GPa, respectively, due to better stress transfer and adhesion between fibre and matrix. Additionally, impact strength at 15 wt. % had maximum value (22 kJ/m^2^), the result showed better matrix interlocking. Water uptake is also lower in banana polyester composite due to fewer voids and uniform distribution [120].

### 7.5. Agave Fibre Composite

Kumar et al. [121] studied the mechanical and wear behaviour of untreated Himalayan agave polymer composite with different sizes (3, 5, and 7 mm) in length and loading (5, 7 and 9 wt. %). The study illustrates that size and loading play a significant role in the composite’s mechanical and wear properties. The maximum tensile and impact strength is achieved at 7 mm fibre length and 9 wt. % of fibre loading. However, flexural strength is maximum at 7 mm and 7 wt. % of fibre in the composite. This study also reveals that parameters like normal load, sliding distance, size, and sliding velocity affect the wear rate and they are decided carefully before a particular application [121]. Mylsamy et al. [122] reported the wear behaviour of agave Americana reinforced with epoxy composites. Agave fibers of lengths 3, 5, and 7 mm are used and fabricated by hand layup and open mould technique. The composite’s wear behaviour is investigated with different parameters, i.e., load, length, and sliding velocity. The result indicated that 3 mm length showed a better wear behaviour when sliding against the stainless steel. The worn surfaces were observed under the scanning electron microscope (SEM). SEM observation indicated that the 3 mm length epoxy composite has a superior bond with the epoxy matrix compared to other lengths. The study suggests that an increase in fibre length in the composite is directly proportional to weight loss and inversely proportional to wear resistance [122]. Effect of alkali treatment and (3, 7, and 10 mm) length on agave/epoxy composites’ mechanical properties reported by Mylsamy et al. [123]. It is treated with 5% NaOH solution at room temperature for 2 days. The result showed that a 3 mm length composite has a better bond with the epoxy resin. Moreover, the composite with NaOH-treated fibres showed better mechanical properties, i.e., tensile, compression, flexural, and impact strength as compared to untreated due to improvement in wetting, impregnation, which result in the mechanical performance. Length of 3 mm was the optimum length for both alkali-treated and untreated as compared to 7 mm and 10 mm length. The result indicated that surface treatment of the fibres is required to moderate mechanical properties as well as better interaction between matrix and filler [123].

**Figure 24:**
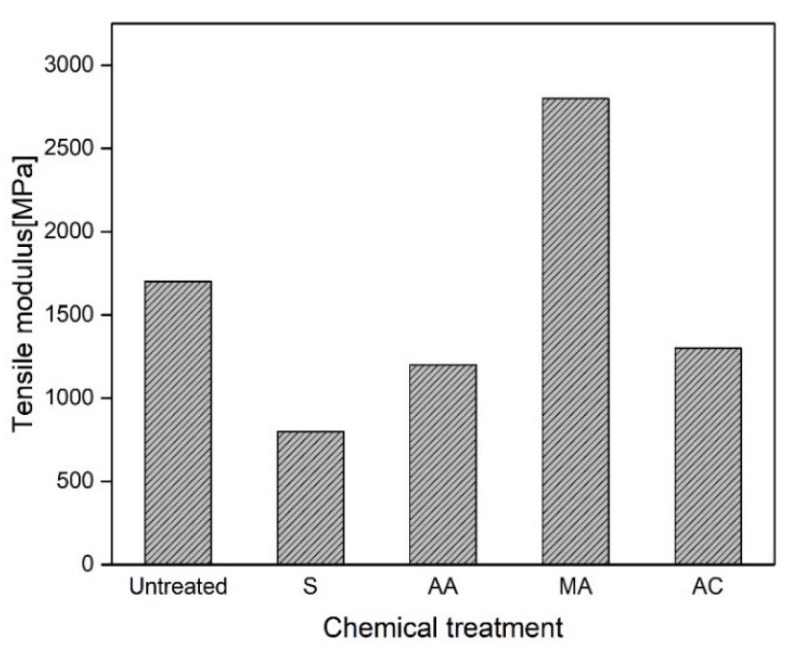
Tensile modulus of untreated and different chemical-treated agaves fibre-reinforced polyester composite. Reprinted with the permission from Reference [125]. Copyright 2008 Elsevier.

Kumar et al. [124] reported the influence of different chemical treatments, i.e., potassium hydroxide, potassium permanganate, and eco-friendly sodium bicarbonate on mechanical and wear properties. The 7 mm length of Himalayan agave was treated with different concentrations, i.e., 5, 10, 15% at 10 wt. % of loading in the polyester matrix. The study exhibited that eco-friendly sodium bicarbonate treatment showed maximum tensile (145 MPa), flexural (214.5 MPa), impact (3.65 J/cm^2^) strength due to better adhesion between treated and polymer, and showed appropriate hardness of 30.33 HV with minimum water absorption capacity as compared to other treated polymer composite. Additionally, sodium bicarbonate-treated Himalayan agave composites showed a lower specific abrasive wear rate [124]. Bessadok et al. [125] studied the effect of chemical modifications such as styrene (S), acetic anhydride (AA), maleic anhydride (MA), and acrylic acid (AC) on mechanical properties and water uptake capacity of agave fiber-reinforced polyester composite. The surface modification studied showed that the water absorption capacity reduces due to a decrease of mass gain, which balances the hydrophobic nature of the matrix and physical changes during the chemical modification. Styrene (S) treatment allows a significant reduction of water uptake capacity. The treated example’s mechanical properties, such as tensile modulus, the breaking strain, and the breaking strength, depending on the chemical treatment used. The tensile modulus of different treatments of agave polyester composite is shown in Figure 24 [125], all the treatments showed a negative impact on the tensile modulus of the surface modification as compared to untreated agave/polyester composite. However, maleic anhydride (MA) showed better results than other chemical treatments due to the removal of some non-cellulosic substances and an increase in the cellulosic component, which enhanced the agave’s mechanical properties in untreated agave. The mechanical properties of different natural fibres are shown in Table 7.

## 8. Prior Studies of Natural Fiber Polymer Composite

**Figure 25:**
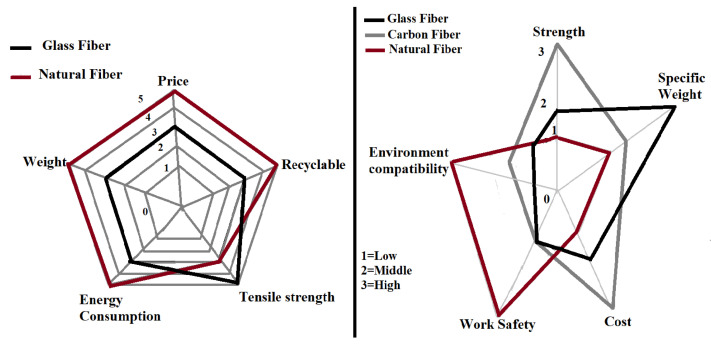
Comparison between natural fibre, glass fibre, and carbon fibre polymer composite. Adapted with the permission from Reference [126]. Copyright 2014 John Wiley and Sons.

Due to environmental aspects, lignocellulosic fibre polymer composite is becoming more popular in use. A modern study estimates that in 2010, globally natural fiber polymer composite material market has the highest value of USD 289.3 million and it can be increased by USD 531.3 million in the next 10 to 12 years [126]. The study also depicts t higher use of natural fibre polymer composite in the automotive sector, becoming the largest market in 2016. The application of natural fiber polymer composite is gaining more preference as compared to synthetic fiber, i.e., carbon fiber and glass fiber. NFC’s has a lack in mechanical strength somehow (strength of synthetic fibre composite is much higher as compared to natural fiber composite). Figure 25 [126], shows the comparison between natural fibre and synthetic fibre polymer composite. The automotive industries adopt natural fiber composite due to its low weight, lower price, biodegradability, and marketing incentives rather than its technical interests. The product range is no longer restricted to non-structural and interior components (door panels or rear shelves). Another impressive trend is seen by using biobased plastic as reinforced matrices for natural fiber composite, and this is gaining attention and approval day by day. The advancement in biobased plastic is attractive from a technological perspective with rapid growth in marketplaces.

The main drawback of natural fiber composite is poor adhesion between the fibre and polymer matrix, and poor water resistance capacity. Therefore, surface modifications of natural fiber are helpful to change the surface properties of the natural fiber to enhance their bond with polymer matrices. Excellent strength and stiffness could be gained with a strong interface that is very brittle in nature with easy propagation of crack through fibre and matrix. The efficiency of stress transfer from the matrix to the fiber can be decreased with a poor interface. Extensive research was carried out and reported in the literature, showing the importance of the interface and the influence of various types of surface modifications on the physical and mechanical properties of natural fiber-reinforced polymer composites. The observed trend indicates a preference for the chemical modification alkaline, silane, acetylation, maleated coupling, permanganate is better as compared to physical modification (corona and plasma treatment). It has also been shown that maleated and silane treatment is becoming a choice method due to beneficial results [127]. Nowadays, chemical links result in an excellent performance to enhance the interface bond between fibres and matrices. A recent study reported by Yue et al. [128], denoting that the enhancement of mechanical properties such as tensile strength, water resistance capability, and thermal stability derived from wasted cotton-seed protein by rational cross-linking and natural fiber, has improved interfacial bonding forces between the fiber and polymer. Additionally, today’s use of enzymes for surface modification of fibre is rapidly increasing. The main reason for the cradling of this technology is the fact that the application of enzymes is environmentally friendly. Moreover, enzyme technology is cost-effective, improves product quality compared to the mostly-used alkali (NaOH), maleated, and has silane modification [129].

## 9. Conclusions and Future Trends

The physical and mechanical properties of composite materials reinforced with natural fibres are discussed in this review paper. Nowadays, leaf-extracted fibre is used in many engineering applications due to its low density, high specific strength, and modulus. Sisal, abaca, pineapple, agave, and banana fibre are potential resource materials for different engineering applications like electrical, automotive, railways, building materials, geotextiles, defense, packaging industry, and household application. The availability and cost of leaf fibre are much lower compared to synthetic fibre. Regarding environmental concerns, the products produced or generated from natural leaf fibre composites are very much eco-friendly with surface modification as compared to synthetic fiber polymer composite. The treatment, like NaOH, sodium bicarbonate (eco-friendly treatment, i.e., non-toxic), and silane-treated fiber, is also an important factor for reducing the water uptake capacity of fiber which is the main drawback in the case of natural fiber-reinforced composite. Chemical treatment helps to enhance the mechanical properties by decreasing the non-cellulosic component in the fibre. It is also notable that excess fiber loading and concertation of treatment can affect the strength of composite due to more fiber content in the matrix and rupture the fiber surface, damaging the primary and secondary wall of fibre. The sustainability of NFC’s environment is higher compared to synthetic fiber due to its biodegradability and renewability ability. Leaf fibre polymer composites are highly energy-efficient and environment friendly because incineration burns completely compared to synthetic fibre, which remains 50% as a residue. Hence, synthetic fibre is harmful to the environment and generates more CO_2_ emissions.

In future use of nano-particles (Aluminium oxide, Silicon carbide, and Graphite), waste from cement and marble industries like fly ash and marble dust, and waste from aluminium industries like red mud used as filler content in bio-resin will help to increase the performance of natural fibre-reinforced polymer composite, helping to replace the synthetic fibre completely. The use of bio-composite and hybrid composite materials in industrial applications can reduce dependence on metal matrix composite. It may also reduce environmental pollution. In-depth research work is required to study the eco-friendly chemical treatment used so that the better performance of composite material reinforced with natural fibres may be utilized in the future.

## Figures and Tables

**Table 1:** Chemical constituent content in different natural fiber [10,39,40,41,42,43].

Category	Fibre Type	Cellulose (wt %)	Hemi-Celluloses (wt %)	Lignin (wt %)
Bast	Flax	71–81	18.6–20.6	2.2–3
	Hemp	57–77	14–22.4	3.7–13
	Nettle	79–86	6.5–12.5	3.5–4.4
	Jute	61–71	14–20	12–13
	Kenaf	28–72	20.3–25	8–22.7
	Roselle	70.2	7.2	14.9
Leaf	Sisal	47–78	10–14.2	7–11
	Abaca	56–63	20–25	7–9
	Henequen	77.6	4–8	13.1
	Pineapple fiber	70.82–81	0–0	5–12.7
	Banana	63–67.6	10–19	5
	Date palm	33.9	26.1	27.7
Fruit/ Seed	Coir (coconut)	32–47	0.15–20	31–45
	Oil palm (EPF) Oil palm (Mesocarp)	65 60	0 0	19 11
	Sponge gourd	50.2–67.2	15.6–21.2	11.2–14.4
	Kapok	64	23	13
	Cotton	82.7–92	5.7–6	0
Grasses	Straw–wheat	28.8–48.8	15–39.1	12–20
	Straw–rice	45	19.3	18.9
	Straw–rye	37.9	36.9	17.6
	Alfa (esparto)	45.4	38.5	14.9
	Bamboo	26–60.8	25.1–30	2.1–32.2
Husk/Hull	Rice husk	35–45	19–25	19.5–20
Sugar	Sugarcane bagasse	36.3–55.2	16.8–24.7	18.14–25.3

**Table 2:** General characteristics of thermoset polymer [2,44].

Characteristics	Thermoset Polymer		
	Polyester Resin	Vinyl-Ester Resin	Epoxy
Density (g/cc)	1.2–1.5	1.2–1.4	1.1–1.4
Elastic modulus (GPa)	2–4.5	3.1–3.8	3–6
Tensile strength (MPa)	40–90	69–83	35–100
Compressive strength (MPa)	90–250	100	100–200
Elongation %	2	4–7	1–6
Cure shrinkage %	4–8	-	1–2
Water absorption (24 h @ 20 °C)	0.1–0.3	0.1	0.1–0.4
Izod impact Notched (J/cm)	0.15–3.2	2.5	0.3

**Table 3:** General characteristics of thermoplastic polymer [44].

Characteristics	Thermoplastic Polymer					
	PP	LDPE	HDPE	PS	Nylon 6	Nylon6,6
Density (g/cc)	0.899–0.920	0.910–0.925	0.94–0.96	1.04–1.06	1.12–1.14	1.13–1.15
Water absorption in 24 h (%)	0.01–0.02	<0.015	0.01–0.2	0.03–0.10	1.3–1.8	1.0–1.6
T_g_ (°C)	–10 to –23	–125	–133 to –100		48	80
T_m_(°C)	160–176	105–116	120–140	110–135	215	250–269
Heat deflection temp (°C)	50–63	32–50	43–60	Max. 220	56–80	75–90
Coefficient of thermal expansion (mm/mm/°C × 10^5^)	6.8–13.5	10	12–13	6–8	8–8.6	7.2–9
Tensile strength (MPa)	26–41.4	40–78	14.5–38	25–69	43–79	12.4–94
Elastic modulus (GPa)	0.95–1.77	0.055–0.38	0.4–1.5	4–5	2.9	2.5–3.9
Izod impact strength (J/cm)	21.4–267	>854	26.7–1.068	1.1	42.7–160	16–654

**Table 4:** Tensile strength of untreated, alkali (NaOH)-treated, and single pineapple leaf fibres (PALF) epoxy composite [99].

PALF	Young’s Modulus (MPa)	Tensile Strength (MPa)	Strain to Failure (%)
Untreated	12.58	532.74	4.83
Alkali treated	15.72	635.44	4.38
Epoxy-coated PALF	14.33	534.88	3.86

**Table 5:** Influence on tensile strength of untreated and different chemical-treated banana fibre-reinforced polyester composite [113].

Composite	Tensile Strength [MPa]
Untreated	57
0.5 NaOH	65
1% NaOH	70
A174	60
A151	61
A1100	58
F8261	48
Si69	45

**Table 6:** Influence on the impact strength of untreated and different chemical-treated banana fibre-reinforced polyester composite [113].

Composite	Impact Strength [kJ/m^2^]
Gum	8
Untreated	14
0.5 NaOH	7.5
1% NaOH	37
A174	28
A151	26
A1100	24
Acetylated	38

**Table 7:** Mechanical properties of different natural fibre [19,26,35,36,39,40,42].

Category	Fibre Type	Density	Tensile Strength (MPa)	Young Modulus (GPa)	Elongation at Break (%)
Bast	Flax	1.4–1.54	88–1500	18–80	1.2–3.2
	Hemp	1.47–1.5	550–690	9.93–70	1.6–4.7
	Nettle	-	650–1594	38–87	1–6
	Jute	1.3–1.5	200–800	10–55	1.16–1.8
	Kenaf	0.749–1.45	223–1191	2.86–53	1.6–5.7
	Roselle	-	147–184	2.76	11–15
Leaf	Sisal	1.33–1.5	80–700	1.46–38	2–15
	Abaca	1.5	400–980	12–31.1	2.9–10
	Henequen	1.2	500	13.2	4.8
	Pineapple	1.44–1.5	170–1627	34.5–82.5	0.8–3
	Banana	1.35	529–914	7.7–32	1.5–53
	Date palm	0.92	170–275	5–12	5–10
Fruit/Seed	Coir	1.15–1.45	106–593	1.27–6	159.9
	Oil palm	0.7–1.55	100–400	1–9	8–25
	Cotton	1.5–1.6	287–800	1.1–12.6	3–10
	Kapok	-	-	-	1.2
Grasses	Straw wheat	1.49	59–140	3.7–4.8	-
	Straw rice	-	150–200	3.3–12.5	3.2–4.6
	Alfa	0.89–1.4	188–350	18–25	1.5–5.8
	Bamboo	0.6–1.1	140–441	11–36	1.3–8
Sugar	Sugarcane bagasse	1.25	290	17	-

## Data Availability

The authors confirm that the data supporting the findings of this study are available within the article.

## References

[1] Cheung H.-Y., Ho M.-P., Lau K.-T., Cardona F., Hui D. (2009). Natural fibre-reinforced composites for bioengineering and environmental engineering applications. Compos. Part B Eng..

[2] Holbery J., Houston D. (2006). Natural-fiber-reinforced polymer composites in automotive applications. JOM.

[3] Sanjay M.R., Arpitha G.R., Naik L.L., Gopalakrishna K., Yogesha B. (2016). Applications of Natural Fibers and Its Composites: An Overview. Nat. Resour..

[4] Mohammed L., Ansari M.N.M., Pua G., Jawaid M., Islam M.S. (2015). A Review on Natural Fiber Reinforced Polymer Composite and Its Applications. Int. J. Polym. Sci..

[5] Duan L., Yu W. Review of recent research in nano cellulose preparation and application from jute fibers. Proceedings of the 2016 3rd International Conference on Materials Engineering, Manufacturing Technology and Control.

[6] Burgueño R., Quagliata M.J., Mehta G.M., Mohanty A.K., Misra M., Drzal L.T. (2005). Sustainable Cellular Biocomposites from Natural Fibers and Unsaturated Polyester Resin for Housing Panel Applications. J. Polym. Environ..

[7] Lescher P., Jayaraman K., Bhattacharyya D. (2012). Characterization of water-free thermoplastic starch blends for manufacturing processes. Mater. Sci. Eng. A.

[8] Le Phuong H.A., Ayob N.A.I., Blanford C.F., Rawi N.F.M., Szekely G. (2019). Nonwoven Membrane Supports from Renewable Resources: Bamboo Fiber Reinforced Poly(Lactic Acid) Composites. ACS Sustain. Chem. Eng..

[9] Zhang S., Tanioka A., Okamoto M., Haraoka Y., Hayashi N., Matsumoto H. (2020). High-Quality Nanofibrous Nonwoven Air Filters: Additive Effect of Water-Jet Nanofibrillated Celluloses on Their Performance. ACS Appl. Polym. Mater..

[10] Fuqua M.A., Huo S., Ulven C.A. (2012). Natural Fiber Reinforced Composites. Polym. Rev..

[11] Prasad L., Singh G., Yadav A., Kumar V., Kumar A. (2019). Properties of functionally gradient composites reinforced with waste natural fillers. Acta Period. Technol..

[12] Begum K., Islam M.A. (2019). Treatment of Shear Stress versus Shear Rate Data for Natural Fiber Reinforced Polymer Composites: A Discussion. J. Sci. Res..

[13] Prasad L., Kumar S., Patel R.V., Yadav A., Kumar V., Winczek J. (2020). Physical and Mechanical Behaviour of Sugarcane Bagasse Fibre-Reinforced Epoxy Bio-Composites. Materials.

[14] Dittenber D.B., GangaRao H.V. (2012). Critical review of recent publications on use of natural composites in infrastructure. Compos. Part A Appl. Sci. Manuf..

[15] Patel M., Narayan R., Mohanty A.K., Misra M., Drzal L.T. (2005). How Sustainable Are Biopolymers and Biobased Products? The Hope, the Doubts, and the Reality. Natural Fibers, Biopolymers, and Biocomposites.

[16] Hanana F.E., Rodrigue D. (2015). Rotational Molding of Polymer Composites Reinforced with Natural Fibers. Plast. Eng..

[17] Santulli C., Sarasini F., Puglia D., Kenny J.M. (2017). 8 Injection moulding of plant fibre composites. Advanced Composite Materials: Properties and Applications.

[18] Chaitanya S., Singh I. (2017). Processing of PLA / Sisal Fiber Biocomposites Using Direct and Extrusion-Injection Molding. Mater. Manuf. Process..

[19] Zini E., Scandola M. (2011). Green composites: An overview. Polym. Compos..

[20] Joshi S.V., Drzal L.T., Mohanty A.K., Arora S. (2004). Are natural fiber composites environmentally superior to glass fiber reinforced composites? Compos. Part A Appl. Sci. Manuf..

[21] Corbière-Nicollier T., Laban B.G., Lundquist L., Leterrier Y., Månson J.-A., Jolliet O. (2001). Life cycle assessment of biofibres replacing glass fibres as reinforcement in plastics. Resour. Conserv. Recycl..

[22] Haylock R., Rosentrater K.A. (2017). Cradle-to-Grave Life Cycle Assessment and Techno-Economic Analysis of Polylactic Acid Composites with Traditional and Bio-Based Fillers. J. Polym. Environ..

[23] Stagner J.A., Tseng S., Tam E.K.L. (2012). Bio-Based Polymers and End-of-Life Vehicles. J. Polym. Environ..

[24] Mohanty A., Misra M., Drzal L. (2018). Sustainable Bio-composites from Renewable Resources: Opportunities and Challenges in the Green Materials World. Renew. Energy.

[25] Kamath S.S., Sampathkumar D., Bennehalli B. (2017). A review on natural areca fibre reinforced polymer composite materials. Ciência Tecnol. Mater..

[26] Faruk O., Bledzki A.K., Fink H.-P., Sain M. (2012). Biocomposites reinforced with natural fibers: 2000–2010. Prog. Polym. Sci..

[27] Prasad L., Singh V., Patel R.V., Yadav A., Kumar V., Winczek J. (2021). Physical and Mechanical Properties of Rambans (Agave) Fiber Reinforced with Polyester Composite Materials. J. Nat. Fibers.

[28] Prasad L., Kumain A., Patel R.V., Yadav A., Winczek J. (2020). Physical and Mechanical Behavior of Hemp and Nettle Fiber-Reinforced Polyester Resin-based Hybrid Composites. J. Nat. Fibers.

[29] Li Y., Mai Y.-W., Ye L. (2000). Sisal fibre and its composites: A review of recent developments. Compos. Sci. Technol..

[30] Chand N., Hashmi S.A.R. (1993). Mechanical properties of sisal fibre at elevated temperatures. J. Mater. Sci..

[31] Idicula M., Joseph K., Thomas S. (2010). Mechanical Performance of Short Banana/Sisal Hybrid Fiber Reinforced Polyester Composites. J. Reinf. Plast. Compos..

[32] Idicula M., Neelakantan N.R., Oommen Z., Joseph K., Thomas S. (2005). A study of the mechanical properties of randomly oriented short banana and sisal hybrid fiber reinforced polyester composites. J. Appl. Polym. Sci..

[33] Shih Y.-F., Cai J.-X., Kuan C.-S., Hsieh C.-F. (2012). Plant fibers and wasted fiber/epoxy green composites. Compos. Part B Eng..

[34] Gholampour A., Ozbakkaloglu T. (2020). A review of natural fiber composites: Properties, modification and processing techniques, characterization, applications. J. Mater. Sci..

[35] Basu P. (2010). Pyrolysis and Torrefaction. Biomass Gasification Design Handbook.

[36] Bledzki A. (1999). Composites reinforced with cellulose based fibres. Prog. Polym. Sci..

[37] John M.J., Thomas S. (2008). Biofibres and biocomposites. Carbohydr. Polym..

[38] Mohanty A.K., Misra M., Hinrichsen G. (2000). Biofibres, biodegradable polymers and biocomposites: An overview. Macromol. Mater. Eng..

[39] Li X., Tabil L.G., Panigrahi S. (2007). Chemical Treatments of Natural Fiber for Use in Natural Fiber-Reinforced Composites: A Review. J. Polym. Environ..

[40] Malkapuram R., Kumar V. (2009). Yuvraj Singh Negi Recent Development in Natural Fiber Reinforced Polypropylene Composites. J. Reinf. Plast. Compos..

[41] Saheb D.N., Jog J.P. (1999). Natural fiber polymer composites: A review. Adv. Polym. Technol..

[42] John M.J., Anandjiwala R.D. (2008). Recent developments in chemical modification and characterization of natural fiber-reinforced composites. Polym. Compos..

[43] Singh C.P., Patel R.V., Hasan M.F., Yadav A., Kumar V., Kumar A. (2021). Fabrication and evaluation of physical and mechanical properties of jute and coconut coir reinforced polymer matrix composite. Mater. Today Proc..

[44] Ku H., Wang H., Pattarachaiyakoop N., Trada M. (2011). A review on the tensile properties of natural fiber reinforced polymer composites. Compos. Part B Eng..

[45] Chung D.D. (2017). Polymer-Matrix Composites: Structure and Processing. Carbon Composites.

[46] Boisse P., Philippe B. (2015). Advances in Composites Manufacturing and Process Design.

[47] Balasubramanian K., Sultan M.T., Rajeswari N. (2018). Manufacturing techniques of composites for aerospace applications. Sustainable Composites for Aerospace Applications.

[48] Gunge A., Koppad P.G., Nagamadhu M., Kivade S., Murthy K.S. (2019). Study on mechanical properties of alkali treated plain woven banana fabric reinforced biodegradable composites. Compos. Commun..

[49] Elkington M., Bloom D., Ward C., Chatzimichali A., Potter K. (2015). Hand layup: Understanding the manual process. Adv. Manuf. Polym. Compos. Sci..

[50] Jamir M.R., Majid M.S., Khasri A. (2018). Natural lightweight hybrid composites for aircraft structural applications. Sustainable Composites for Aerospace Applications.

[51] Perna A.S., Viscusi A., Astarita A., Boccarusso L., Carrino L., Durante M., Sansone R. (2019). Manufacturing of a Metal Matrix Composite Coating on a Polymer Matrix Composite Through Cold Gas Dynamic Spray Technique. J. Mater. Eng. Perform..

[52] Marques A. (2011). Fibrous materials reinforced composites production techniques. Fibrous and Composite Materials for Civil Engineering Applications.

[53] Ervina J., Ghaleb Z., Hamdan S., Mariatti M. (2019). Colloidal stability of water-based carbon nanotube suspensions in electrophoretic deposition process: Effect of applied voltage and deposition time. Compos. Part A Appl. Sci. Manuf..

[54] Rajak D.K., Pagar D.D., Menezes P.L., Linul E. (2019). Fiber-Reinforced Polymer Composites: Manufacturing, Properties, and Applications. Polymers.

[55] Yalcinkaya M.A., Guloglu G.E., Pishvar M., Amirkhosravi M., Sozer E.M., Altan M.C., Sozer M. (2019). Pressurized Infusion: A New and Improved Liquid Composite Molding Process. J. Manuf. Sci. Eng..

[56] Plummer C.J.G., Bourban P.-E., Månson J.-A. (2016). Polymer Matrix Composites: Matrices and Processing. Reference Module in Materials Science and Materials Engineering.

[57] Mitschang P., Hildebrandt K. (2012). Polymer and composite moulding technologies for automotive applications. Advanced Materials in Automotive Engineering.

[58] Park C.H., Lee W.I. (2012). Compression molding in polymer matrix composites. Manufacturing Techniques for Polymer Matrix Composites (PMCs).

[59] Matveenko V.P., Kosheleva N.A., Shardakov I.N., Voronkov A.A. (2018). Temperature and strain registration by fibre-optic strain sensor in the polymer composite materials manufacturing. Int. J. Smart Nano Mater..

[60] Correia J.R. (2013). Pultrusion of advanced fibre-reinforced polymer (FRP) composites. Advanced Fibre-Reinforced Polymer (FRP) Composites for Structural Applications.

[61] Verma D., Joshi G., Dabral R., Lakhera A. (2019). Processing and evaluation of mechanical properties of epoxy-filled E-glass fiber–fly ash hybrid composites. Mechanical and Physical Testing of Biocomposites, Fibre-Reinforced Composites and Hybrid Composites.

[62] Joshi S. (2012). The pultrusion process for polymer matrix composites. Manufacturing Techniques for Polymer Matrix Composites (PMCs).

[63] Bhardwaj N., Kundu S.C. (2010). Electrospinning: A fascinating fiber fabrication technique. Biotechnol. Adv..

[64] Wang G., Yu D., Kelkar A.D., Zhang L. (2017). Electrospun nanofiber: Emerging reinforcing filler in polymer matrix composite materials. Prog. Polym. Sci..

[65] González-Henríquez C.M., Sarabia-Vallejos M.A., Rodriguez-Hernandez J. (2019). Polymers for additive manufacturing and 4D-printing: Materials, methodologies, and biomedical applications. Prog. Polym. Sci..

[66] Chua C.K., Leong K.F. (2017). 3D Printing and Additive Manufacturing.

[67] Mantell S.C., Springer G.S. (1994). Filament winding process models. Compos. Struct..

[68] Minsch N., Herrmann F., Gereke T., Nocke A., Cherif C. (2017). Analysis of Filament Winding Processes and Potential Equipment Technologies. Procedia CIRP.

[69] Hopmann C., Wruck L., Schneider D., Fischer K. (2019). Automated Winding of Preforms Directly from Roving. Light. Des. Worldw..

[70] Pickering K.L., Efendy M.G.A., Le T.M. (2016). A review of recent developments in natural fibre composites and their mechanical performance. Compos. Part A Appl. Sci. Manuf..

[71] Rong M.Z., Zhang M.Q., Liu Y., Yang G.C., Zeng H.M. (2001). The effect of fiber treatment on the mechanical properties of unidirectional sisal-reinforced epoxy composites. Compos. Sci. Technol..

[72] Nimanpure S., Hashmi S., Kumar R., Nigrawal A., Bhargaw H., Naik A. (2017). Sisal fibril epoxy composite-a high strength electrical insulating material. Polym. Compos..

[73] Maya M., George S.C., Jose T., Sreekala M., Thomas S. (2017). Mechanical Properties of Short Sisal Fibre Reinforced Phenol Formaldehyde Eco-Friendly Composites. Polym. Renew. Resour..

[74] Li Y., Ma H., Shen Y., Li Q., Zheng Z. (2015). Effects of resin inside fiber lumen on the mechanical properties of sisal fiber reinforced composites. Compos. Sci. Technol..

[75] Betelie A.A., Megera Y.T., Redda D.T., Sinclair A. (2018). Experimental investigation of fracture toughness for treated sisal epoxy composite. AIMS Mater. Sci..

[76] Oksman K., Skrifvars M., Selin J.-F. (2003). Natural fibres as reinforcement in polylactic acid (PLA) composites. Compos. Sci. Technol..

[77] Sreekumar P., Thomas S.P., Saiter J.M., Joseph K., Unnikrishnan G., Thomas S. (2009). Effect of fiber surface modification on the mechanical and water absorption characteristics of sisal/polyester composites fabricated by resin transfer molding. Compos. Part A Appl. Sci. Manuf..

[78] Sreekumar P., Joseph K., Unnikrishnan G., Thomas S. (2007). A comparative study on mechanical properties of sisal-leaf fibre-reinforced polyester composites prepared by resin transfer and compression moulding techniques. Compos. Sci. Technol..

[79] Singh B., Gupta M., Verma A. (1996). Influence of fiber surface treatment on the properties of sisal-polyester composites. Polym. Compos..

[80] Sangthong S., Pongprayoon T., Yanumet N. (2009). Mechanical property improvement of unsaturated polyester composite reinforced with admicellar-treated sisal fibers. Compos. Part A Appl. Sci. Manuf..

[81] Khanam P.N., Khalil H.P.S.A., Reddy G.R., Naidu S.V. (2011). Tensile, Flexural and Chemical Resistance Properties of Sisal Fibre Reinforced Polymer Composites: Effect of Fibre Surface Treatment. J. Polym. Environ..

[82] Liu K., Zhang X., Takagi H., Yang Z., Wang D. (2014). Effect of chemical treatments on transverse thermal conductivity of unidirectional abaca fiber/epoxy composite. Compos. Part A Appl. Sci. Manuf..

[83] Cai M., Takagi H., Nakagaito A.N., Li Y., Waterhouse G.I. (2016). Effect of alkali treatment on interfacial bonding in abaca fiber-reinforced composites. Compos. Part A Appl. Sci. Manuf..

[84] Shibata M., Takachiyo K.-I., Ozawa K., Yosomiya R., Takeishi H. (2002). Biodegradable polyester composites reinforced with short abaca fiber. J. Appl. Polym. Sci..

[85] Punyamurthy R., Sampathkumar D., Srinivasa C.V., Bennehalli B. (2012). Effect of alkali treatment on water absorption of single cellulosic abaca fiber. BioResources.

[86] Punyamurthy R., Sampathkumar D., Bennehalli B., Srinivasa C.V. (2014). Abaca fiber reinforced epoxy composites: Evaluation of impact strength. Int. J. Sci. Basic Appl..

[87] Paglicawan M.A., Basilia B.A., Kim B.S. (2013). Water Uptake and Tensile Properties of Plasma Treated Abaca Fiber Reinforced Epoxy Composite. Compos. Res..

[88] Malenab R.A.J., Ngo J.P.S., Promentilla M.A.B. (2017). Chemical Treatment of Waste Abaca for Natural Fiber-Reinforced Geopolymer composite. Materials.

[89] Ahmed S.N., Prabhakar M.N., Siddaramaiah, Song J.I. (2017). Influence of silane-modified Vinyl ester on the properties of Abaca fiber reinforced composites. Adv. Polym. Technol..

[90] Liu Y., Ma Y., Yu J., Zhuang J., Wu S., Tong J. (2018). Development and characterization of alkali treated abaca fiber reinforced friction composites. Compos. Interfaces.

[91] Batara A.G.N., Llanos P.S.P., De Yro P.A.N., Sanglay G.C.D., Magdaluyo E.R. (2019). Surface modification of abaca fibers by permanganate and alkaline treatment via factorial design. AIP Conf. Proc..

[92] Devi L.U., Bhagawan S.S., Thomas S. (1997). Mechanical properties of pineapple leaf fiber-reinforced polyester composites. J. Appl. Polym. Sci..

[93] Mishra S., Misra M., Tripathy S.S., Nayak S.K., Mohanty A.K. (2001). Potentiality of Pineapple Leaf Fibre as Reinforcement in PALF-Polyester Composite: Surface Modification and Mechanical Performance. J. Reinf. Plast. Compos..

[94] Devi L.U., Joseph K., Nair K.C.M., Thomas S. (2004). Ageing studies of pineapple leaf fiber-reinforced polyester composites. J. Appl. Polym. Sci..

[95] Senthilkumar K., Saba N., Chandrasekar M., Jawaid M., Rajini N., Alothman O.Y., Siengchin S. (2019). Evaluation of mechanical and free vibration properties of the pineapple leaf fibre reinforced polyester composites. Constr. Build. Mater..

[96] Senthilkumar K., Rajini N., Saba N., Chandrasekar M., Jawaid M., Siengchin S. (2019). Effect of Alkali Treatment on Mechanical and Morphological Properties of Pineapple Leaf Fibre/Polyester Composites. J. Polym. Environ..

[97] Krishnasamy S., Muthukumar C., Nagarajan R., Thiagamani S.M.K., Saba N., Jawaid M., Siengchin S., Ayrilmis N. (2019). Effect of fibre loading and Ca(OH)2 treatment on thermal, mechanical, and physical properties of pineapple leaf fibre/polyester reinforced composites. Mater. Res. Express.

[98] Devi L.U., Bhagawan S., Thomas S. (2011). Dynamic mechanical properties of pineapple leaf fiber polyester composites. Polym. Compos..

[99] Payae Y., Lopattananon N. (2009). Adhesion of pineapple-leaf fiber to epoxy matrix: The role of surface treatments. Songklanakarin J. Sci. & Technol..

[100] Lopattananon N., Payae Y., Seadan M. (2008). Influence of fiber modification on interfacial adhesion and mechanical properties of pineapple leaf fiber-epoxy composites. J. Appl. Polym. Sci..

[101] Lopattananon N., Panawarangkul K., Sahakaro K., Ellis B. (2006). Performance of pineapple leaf fiber–natural rubber composites: The effect of fiber surface treatments. J. Appl. Polym. Sci..

[102] Reddy M.I., Varma U.P., Kumar I.A., Manikanth V., Raju P.K. (2018). Comparative Evaluation on Mechanical Properties of Jute, Pineapple leaf fiber and Glass fiber Reinforced Composites with Polyester and Epoxy Resin Matrices. Mater. Today Proc..

[103] Mangal R., Saxena N., Sreekala M., Thomas S., Singh K. (2003). Thermal properties of pineapple leaf fiber reinforced composites. Mater. Sci. Eng. A.

[104] Mohamed A.R., Sapuan S.M., Shahjahan M., Khalina A., Sapuan M.S. (2010). Effects of Simple Abrasive Combing and Pretreatments on the Properties of Pineapple Leaf Fibers (Palf) and Palf-Vinyl Ester Composite Adhesion. Polym. Technol. Eng..

[105] Mohamed A.R., Sapuan S.M., Khalina A., Sapuan M.S. (2014). Mechanical and thermal properties of josapine pineapple leaf fiber (PALF) and PALF-reinforced vinyl ester composites. Fibers Polym..

[106] Jayaseelan C., Padmanabhan P., Athijayamani A., Ramanathan K. (2017). Comparative Investigation of Mechanical Properties of Epoxy Composites Reinforced with Short Fibers, Macro Particles, and Micro Particles. Bioresources.

[107] Prasad S.V.N.B., Krishna M.R.R., Ashok A., Aryan A., Krishna T.H.G. (2018). Study of mechanical and water absorption characteristics of natural fibre reinforced epoxy composites. IOP Conf. Ser. Mater. Sci. Eng..

[108] Mohan T., Kanny K. (2019). Compressive characteristics of unmodified and nanoclay treated banana fiber reinforced epoxy composite cylinders. Compos. Part B Eng..

[109] Venkateshwaran N., Perumal A.E., Arunsundaranayagam D. (2013). Fiber surface treatment and its effect on mechanical and visco-elastic behaviour of banana/epoxy composite. Mater. Des..

[110] Venkateshwaran N., Elayaperumal A., Jagatheeshwaran M.S. (2011). Effect of fiber length and fiber content on mechanical properties of banana fiber/epoxy composite. J. Reinf. Plast. Compos..

[111] Yadav S.K.J., Vedrtnam A., Gunwant D. (2020). Experimental and numerical study on mechanical behavior and resistance to natural weathering of sugarcane leave reinforced polymer composite. Constr. Build. Mater..

[112] Venkateshwaran N., ElayaPerumal A., Raj R.H.A. (2012). Mechanical and Dynamic Mechanical Analysis of Woven Banana/Epoxy Composite. J. Polym. Environ..

[113] Pothan L.A., George J., Thomas S. (2002). Effect of fiber surface treatments on the fiber–matrix interaction in banana fiber reinforced polyester composites. Compos. Interfaces.

[114] Maleque M., Yousif B., Sapuan S. (2007). Mechanical properties study of pseudo-stem banana fiber reinforced epoxy composite. Arab. J. Sci. Eng..

[115] Shivamurthy B., Thimmappa B.H.S., Monteiro J. (2020). Sliding wear, mechanical, flammability, and water intake properties of banana short fiber/Al(OH)3/epoxy composites. J. Nat. Fibers.

[116] Bharadiya P.S., Singh M.K., Mishra S. (2018). Influence of Graphene Oxide on Mechanical and Hydrophilic Properties of Epoxy/Banana Fiber Composites. JOM.

[117] Pothan L.A., Thomas S., Neelakantan N.R. (1997). Short Banana Fiber Reinforced Polyester Composites: Mechanical, Failure and Aging Characteristics. J. Reinf. Plast. Compos..

[118] Ike-Eze I.C.E., Uyor U.O., Aigbodion V.S., Omah A.D., Ude S.N., Daniel-Nkpume C.C., Ike-Eze I.C.E. (2019). Tensile and compressive strength of palm kernel shell particle reinforced polyester composites. Mater. Res. Express.

[119] Karthikeyan M.K.V., Balaji A.N., Vignesh V. (2016). Effect of rope mat and random orientation on mechanical and thermal properties of banana ribbon-reinforced polyester composites and its application. Int. J. Polym. Anal. Charact..

[120] Mariatti M., Jannah M., Abu Bakar A., Khalil H.A. (2008). Properties of Banana and Pandanus Woven Fabric Reinforced Unsaturated Polyester Composites. J. Compos. Mater..

[121] Kumar S., Prasad L., Kumar S., Patel V.K. (2019). Physico-mechanical and Taguchi-designed sliding wear properties of Himalayan agave fiber reinforced polyester composite. J. Mater. Res. Technol..

[122] Mylsamy K., Rajendran I. (2011). Influence of Fibre Length on the Wear Behaviour of Chopped Agave americana Fibre Reinforced Epoxy Composites. Tribol. Lett..

[123] Mylsamy K., Rajendran I. (2011). Influence of alkali treatment and fibre length on mechanical properties of short Agave fibre reinforced epoxy composites. Mater. Des..

[124] Kumar S., Prasad L., Kumar S., Patel V.K. (2020). Physicomechanical and Taguchi optimized abrasive wear behaviour of KOH/KMnO4/NaHCO3 treated Himalayan Agave fiber reinforced polyester composite. Mater. Res. Express.

[125] Bessadok A., Marais S., Roudesli S., Lixon C., Métayer M. (2008). Influence of chemical modifications on water-sorption and mechanical properties of Agave fibres. Compos. Part A Appl. Sci. Manuf..

[126] Faruk O., Bledzki A.K., Fink H.-P., Sain M. (2014). Progress Report on Natural Fiber Reinforced Composites. Macromol. Mater. Eng..

[127] Xie Y., Hill C.A., Xiao Z., Militz H., Mai C. (2010). Silane coupling agents used for natural fiber/polymer composites: A review. Compos. Part A Appl. Sci. Manuf..

[128] Yue H., Zheng Y., Zheng P., Guo J., Fernández-Blázquez J.P., Clark J.H., Cui Y. (2020). On the improvement of properties of bioplastic composites derived from wasted cottonseed protein by rational cross-linking and natural fiber reinforcement. Green Chem..

[129] Bledzki A.K., Mamun A.A., Jaszkiewicz A., Erdmann K. (2010). Polypropylene composites with enzyme modified abaca fibre. Compos. Sci. Technol..

